# Multiparametric functional characterization of individual lipid nanoparticles using surface-sensitive light-scattering microscopy

**DOI:** 10.1073/pnas.2426601122

**Published:** 2025-05-22

**Authors:** Mattias Sjöberg, Erik Olsén, Mokhtar Mapar, Petteri Parkkila, Simon Niederkofler, Sara Mohammadi, Yujia Jing, Gustav Emilsson, Lennart Lindfors, Björn Agnarsson, Fredrik Höök

**Affiliations:** ^a^Division of Nano and Biophysics, Department of Physics, Chalmers University of Technology, Gothenburg 412 96, Sweden; ^b^Nanolyze, Gothenburg 431 83, Sweden; ^c^Advanced Drug Delivery, Pharmaceutical Sciences, R&D, AstraZeneca, Gothenburg 431 83, Sweden

**Keywords:** light scattering, fluorescence, optical microscopy, lipid nanoparticles, mRNA

## Abstract

The most efficient lipid nanoparticles (LNPs) for gene therapeutics rely on specific lipids that protect the oligonucleotide cargo and aid cellular uptake and subsequent endosomal escape. Yet, the efficacy of current state-of-the-art LNP formulations remains low, a few percent at best. A deeper understanding of how LNP cargo, lipid composition, stoichiometry, size, structure, and pH-induced conformational changes influence their efficiency is therefore necessary for improved design. Given the variability of these properties, preferred screening methods should offer single-particle-resolved multiparametric characterization. In this work, we employ combined surface-sensitive fluorescence and label-free scattering microscopy with single LNP resolution, which when integrated with microfluidics for liquid exchange between media of varying refractive index, enables quantification of LNP size, refractive index, and cargo content. We investigate two LNP formulations that, while similar in size and mRNA content, exhibit differences in functional mRNA delivery. Correlating size with the content of Cy5-labeled mRNA revealed that the cargo scaled with LNP volume for both types of LNPs, while the refractive index varied marginally across LNP size. While this multiparametric fingerprinting alone could not distinguish the two LNP formulations, we use the same experimental platform to show that their difference in fusogenicity to a supporting lipid bilayer under early endosomal conditions (drop in pH from 7.4 to 6.0) correlates with observed differences in in vitro cellular data. This highlights a limitation of the current state-of-the-art toolbox for in situ LNP characterization, which generally focuses on structural properties of suspended LNPs, which may not adequately capture functional performance.

## Significance

By integrating surface-sensitive fluorescence and label-free scattering microscopy with microfluidics, we present an approach to lipid nanoparticle (LNP) characterization that links physicochemical properties such as size, refractive index, and payload content-with functional behavior at the single-LNP level. Our findings reveal that fusogenic behavior of LNPs under endosomal conditions, modeled using a supported lipid bilayer (SLB), cannot be predicted solely from multiparametric physicochemical characterization. This methodology thus addresses a critical bottleneck in LNP-based analytics which could help bridge the gap between LNP properties in suspension and their performance in cellular environments, ultimately guiding the design of more effective LNP therapeutics.

Biological and synthetic nanoparticles show great promise in new drug delivery applications, particularly in the clinical translation of RNA therapeutics (1). In this context, lipid nanoparticles (LNPs), typically made from ionizable cationic lipids, helper lipids, and poly(ethylene glycol) (PEG) lipids (2, 3), have emerged as particularly promising candidates, as demonstrated by their successful implementation as mRNA carriers for COVID-19 vaccines (4, 5). However, the functional delivery of oligonucleotides utilizing even the most efficient LNP formulations is generally low, a few percent at best (6–8), which consequently hampers mRNA translation and limits wide therapeutic use. The efficacy is known to be highly dependent on the properties of the LNPs, as concluded from multiple studies combining in vitro and in vivo measurements with prescreening of different LNP batches with respect to for example size (9–12), zeta-potential (13), cargo encapsulation (9), surface characteristics (14), and structural properties (15–17). Furthermore, extensive theoretical work has also highlighted how both structural features and lipid composition may influence LNP uptake and subsequent translocation of functional genetic material across the endosomal membrane of cells (18–21).

LNP properties are typically controlled by varying the cargo content, lipid composition, and formulation buffer to for example control the size (12, 22, 23). In addition to varying the size to influence cellular uptake and processing (24), the function of LNPs has also been shown to depend on structure (12, 20, 21), which in turn depends on the lipid composition and the relative ratio of the different components (12, 20, 21, 25). For example, LNPs with an ordered bicontinuous cubic internal structure have been shown to deliver siRNA with a higher efficacy than LNPs having a lamellar arrangement (26). Further, it was recently demonstrated that LNPs with high surface coverage of 1,2-distearoyl-*sn*-glycero-3-phosphocholine (DSPC) lipids displayed a strong increase in functional response when the surface coverage of the DSPC was kept constant as the LNP size was varied, while a more than one order of magnitude reduction in functional response was observed if the lipid composition was chosen such that the surface coverage increased with LNP size (12). Strikingly, the protein expression levels in cells exposed to LNPs with a mean diameter of 130 nm differed by up to an order of magnitude depending on these surface characteristics. This observation was attributed to differences in the ability of the LNPs to induce endosomal escape, a hypothesis recently reinforced by in situ measurements of pH-induced LNP fusogenicity with a supported endosomal membrane (27).

It is in this context worthwhile to recall that LNPs are typically prepared using microfluidic-assisted rapid mixing precipitation protocols (25, 28), and although both mean diameter and structure can in this way be controlled by varying cargo and lipid content (22), the full-width-at-half-maximum of the size distribution is often broad and in some cases even comparable to the mean diameter of the LNPs (12, 22). This broad size distribution originates from the complex kinetics of the self-assembly process of lipid–oligonucleotide complexes, characterized by a rapid (subsecond) electrostatically driven association between oligonucleotides and ionizable lipids followed by a slower phase (seconds to minutes) during which rearrangement and condensation result in a kinetically trapped LNP structure (16). As the average LNP size is controlled by the lipid composition, one cannot exclude that structural or compositional variations also exist within individual LNP batches. Consequently, individual LNPs from the same batch may display differences in their capacity to produce a functional biological response. This, in turn, has triggered intense efforts to develop methods capable of providing multiparametric physicochemical characterization of individual LNPs.

Due to their compatibility with near-native liquid environments and high-throughput capability, diverse optical microscopy techniques have emerged as leading tools for characterizing biological nanoparticles with single-nanoparticle resolution. Nanoparticle tracking analysis (NTA) is commonly used to determine the size of suspended nanoparticles via their diffusion constant, by analyzing individual nanoparticle trajectories using either dark-field or fluorescence microscopy (29, 30). However, due to signal fluctuations caused by diffusion of nanoparticles in and out of the illuminated observation volume, quantification of refractive index and cargo content is rather uncertain (30, 31). This can be circumvented by confining suspended LNPs in submicrometer wells, which offers more stable fluorescence emission signals from which cargo content can be better correlated with size (32). Another strategy is to use high framerate imaging methods to minimize the statistical uncertainty during measurements, which was recently combined with interferometric scattering microscopy (iSCAT) to provide precise quantification of both refractive index and size of suspended nanoparticles (33, 34), as verified with extracellular vesicles. Other emerging technologies that have been widely applied to study biological nanoparticles include nanoflow cytometry (35) and single-particle trapping for fluorescence (36) and Raman (37) analysis. The first two methods provide high-throughput measurements on single nanoparticle sizes versus fluorescence content, while the latter offers label-free chemical fingerprinting of individual trapped nanoparticles in solution.

While the methods above cover a wide parameter space for biological characterization, the fact that they are all suspension-based complicates the ability to capture temporally resolved changes at the single-particle level, i.e. tracking how parameters such as size, mass, refractive index, and fluorescence varies over time and relates to particle function such as fusogenicity with a lipid bilayer. Measuring properties of single particles over time is however rather straightforward using surface-sensitive evanescent-light imaging (38), either for time-resolved total internal reflection fluorescence (TIRF) microscopy (39), or in label-free light-scattering-based characterization of faint stationary surface-bound biological nanoparticles (38). The latter approach can also be combined with simultaneous fluorescent readout as well as microfluidic liquid handling, which enable temporal monitoring of surface-bound nanoparticles in different media to reveal, for example, information on biomolecular and nanoparticle binding kinetics (40) and/or structural changes (41). However, the determination of nanoparticle size based on the scattering signal alone is complicated due to the requirement of some form of internal intensity calibration and prior knowledge of the nanoparticles’ refractive index (42).

This limitation in single-particle multiparametric profiling using surface sensitive microscopy is here overcome by using simultaneous waveguide-based fluorescence and label-free light-scattering imaging. By combining this with microfluidic-assisted variation in the refractive index of the media surrounding the nanoparticles, it is possible to quantify size, refractive index, and fluorescent cargo content of biological nanoparticles (43), which was here applied on individual LNPs bound to a supported lipid bilayer (SLB). Additionally, this approach makes it possible to control the surrounding pH, enabling the ability to evaluate how particle properties of individual LNPs relate to the pH-induced fusion with the SLB to mimic the endosomal escape step, as previously demonstrated using TIRF microscopy (27).

The study focused on two distinct types of LNPs, both of which were formulated using *O*-(Z,Z,Z,Z-heptatriaconta-6,9,26,29-tetraem-19-yl)-4-(*N*,*N*-dimethylamino)butanoate (DLin-MC3-DMA), DSPC, cholesterol, and 1,2-dimyristoyl-sn-glycero-3-phosphoethanolamine-N- [methoxy(polyethyleneglycol)-2000] (DMPE-PEG) but having different molar ratios of DSPC to DMPE-PEG, which is known to have a significant effect on the functional mRNA delivery in recipient cells (12, 20). We henceforth refer to these two LNP formulations as low-DSPC LNPs (0.7 mol-% DMPE-PEG and 4.6 mol-% DSPC) and high-DSPC LNPs (0.25 mol-% DMPE-PEG and 10 mol-% DSPC), the former of which has been shown to display a markedly higher transfection efficiency (12). To enable combined scattering and fluorescence inspection, 20 wt-% of the mRNA content was Cy5-labeled (Cy5-mRNA) and 0.06 mol-% of the lipid content was Rhodamine-labeled (Rhod-DOPE). In addition, 0.006 mol-% biotinylated lipids (DSPE-PEG-Biotin) were included to enable specific immobilization to a streptavidin functionalized SLB formed on the surface of the waveguide chip, thus mimicking the binding mechanism between protein coronated LNPs and cell membrane receptors that is believed to translate to LNP attachment to the endosomal membrane upon endocytosis (44). The two types of LNPs display significant differences in their capacity to undergo pH-induced fusion with the SLB; yet, they could not be distinguished based on differences in LNP size, refractive index, and fluorescent cargo content, and no correlations were found between these properties and the fusogenicity. These findings emphasize the need for single-particle multiparametric characterization that extends beyond size, refractive index, and cargo content.

## Results and Discussions

**Fig. 1.**
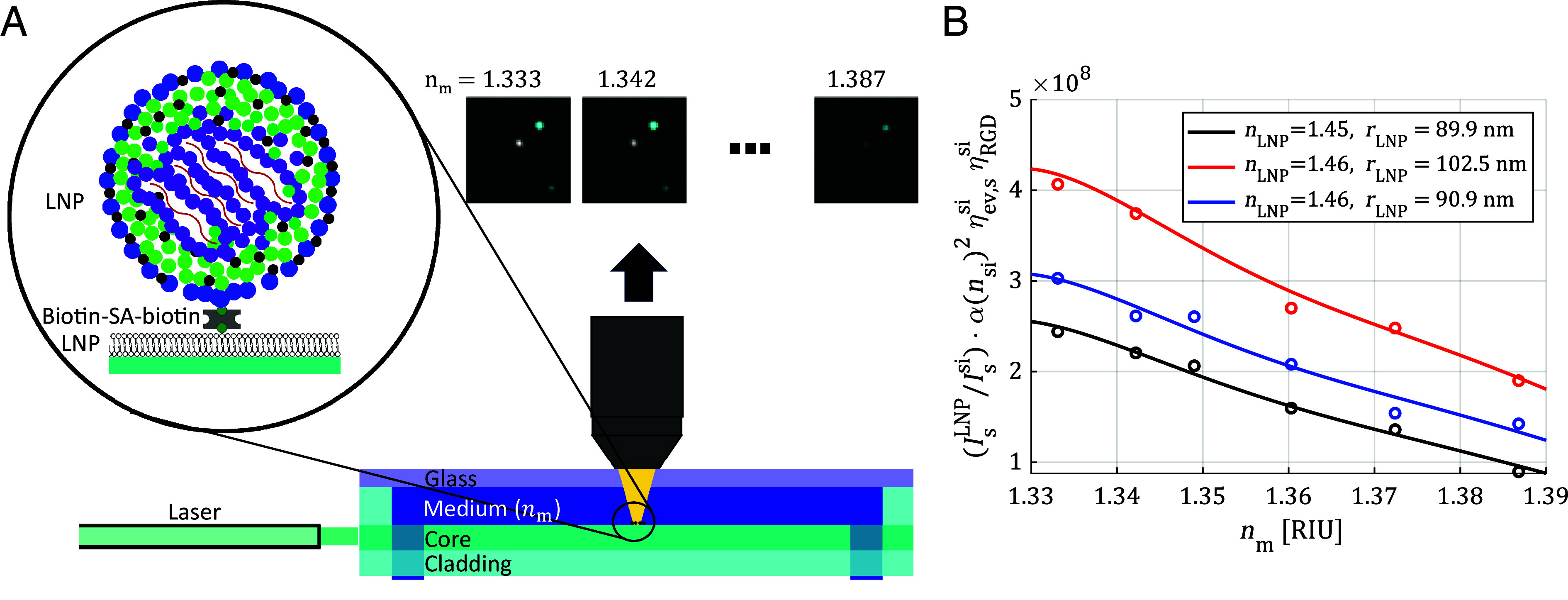
(*A*) Schematic illustration displaying the measurement configuration of the waveguide microscopy setup (Nanolyze™). A water-immersion objective is placed above the measurement area of the waveguide chip and the resulting image projected onto a W-View Gemini image-splitter (Hamamatsu Inc.) allowing single surface-bound particles to be monitored simultaneously in both scattering and fluorescence mode in media of different refractive index or different pH. Lipid nanoparticles (LNPs, depicted in blue in the micrographs) are specifically bound to a supported lipid bilayer (SLB) on the surface using biotin–streptavidin linking (see left schematic) while reference silica particles (depicted in gray in micrographs) are electrostatically adsorbed to the surface. The scattering intensity is subsequently recorded in media with varying refractive indices ($n_{m}$). Details of the waveguide structure are discussed elsewhere (45) (*B*). Examples of the measured scattering intensity ($I_{s}^{LNP}$) for three individual LNPs (circles), normalized to the scattering intensities of the reference NPs and its associated correction factors $I_{s}^{Si}{\left|\alpha \left(n_{Si}\right)\right|}^{2}\eta_{ev,s}^{Si}\eta_{RGD}^{Si}$ (see Eq. **3**), versus medium refractive index ($n_{m}$). The solid curves show the corresponding fits according to Eq. **3** from which $r_{LNP}$ and $n_{LNP}$ were determined.

A schematic of the measurement configuration (Nanolyze™) is presented in Fig. 1*A*. Before binding of biotin-modified LNPs to the streptavidin-modified SLB on the waveguide surface, silica nanoparticles (NPs) with 75 nm radius (SI Appendix, section 1 and Fig. S1), serving as reference particles, were randomly adsorbed to the surface. After an NP coverage of approximately 0.01 NPs/μm^2^ had been achieved, the channel was rinsed and LNPs (20 pM) subsequently injected at a flow rate of 20 μL/min followed by rinsing with PBS buffer when a coverage of around 0.1 LNPs/μm^2^ had been reached (see examples of bound LNPs and NPs in the micrographs in Fig. 1*A* and SI Appendix, section 17 and Movie M1). To correlate the label-free scattering signals from individual LNPs with their Cy5-mRNA and Rhod-DOPE content, complementary fluorescence images were simultaneously recorded using an image splitter [see SI Appendix, section 2 and Fig. S2 and reference (40) for a detailed setup description]. Subsequently, a series of images, using fixed camera exposure and laser intensity, were acquired at different refractive indices of the medium ($n_{m}$) surrounding the particles. This was achieved by sequential injections of PBS buffer solutions of increasing iodixanol concentration (SI Appendix, section 3), which resulted in a gradual decrease in the respective scattering intensities of the surface-bound particles (see micrographs in Fig. 1*A*.)

The quantification of the radius ($r_{LNP}$) and refractive index ($n_{LNP}$) of individual LNPs was obtained by assessing the changes in scattering intensity ($I_{s}$) upon concurrent exchanges of the aqueous medium ($n_{m}$) surrounding the immobilized LNPs and reference NPs. The analysis employed a theoretical model based on the Rayleigh-Gans-Debye (RGD) approximation, where it was assumed that the LNPs could be considered as homogeneous spheres, and analogous to liposomes, impermeable to the iodixanol solutions used for varying $n_{m}$ (46). This approach, summarized below and detailed in the SI Appendix, section 4, enables the extraction of both $r_{LNP}$ and $n_{LNP}$ from the measured scattering intensities of individual LNPs ($I_{s}^{LNP}$) and silica NPs, ($I_{s}^{Si}$).

In brief, the scattering intensity from a particle interacting with an evanescent illumination is given by

[1] $$I_{s}=I_{s}^{0}\eta_{\mathrm{ev},s}\eta_{\mathrm{RGD}},$$

where

[2] $$I_{s}^{0}=A\left(\lambda_{0},n_{m}\right){\left|\alpha \right|}^{2},$$

is the intensity calculated in the Rayleigh limit, α is the polarizability of the particle and $A\left(\lambda_{0}{,n}_{m}\right)$ is a function that includes the square of the illumination field intensity at the surface as well as the capturing efficiency of the optical setup, wavelength dependence, and the sensitivity of the camera, while $\eta_{ev,s}$ and $\eta_{RGD}$ are dimensionless correction factors accounting for the exponentially decaying evanescent field and phase shifts according to the RGD approximation, respectively.

The RGD approximation allows explicit expressions to be derived for $\eta_{RGD}$, which in turn allows measured $I_{s}$ values for individual nanoparticles to be plotted versus $n_{m}$ and the data to be fitted to Eq. **1** using particle size and refractive index as free fitting parameters (45, 47, 48). However, a more practical way of obtaining the parameters is to compare $I_{s}$ to the scattering intensity of a reference particle ($I_{s}^{ref}$) of known radius ($r_{ref}$), and refractive index ($n_{ref}$), and presenting the data as the ratio

[3] $$\frac{I_{s}}{I_{s}^{\mathrm{ref}}}=\frac{{\left|\alpha \left(n\right)\right|}^{2}}{{\left|\alpha \left(n_{\mathrm{si}}\right)\right|}^{2}}\frac{\eta_{\mathrm{ev},s}\eta_{\mathrm{RGD}}}{\eta_{\mathrm{ev},s}^{\mathrm{ref}}\eta_{\mathrm{RGD}}^{\mathrm{ref}}}.$$

This approach eliminates the parameter $A\left(\lambda_{0}{,n}_{m}\right)$, along with its inherent dependence on surface field intensity, variations in the optical configuration, and fluctuations in illumination source intensity.

By normalizing the scattering intensities of individual LNPs to those of silica reference particles, $I_{s}^{Si}$, and plotting $I_{s}/I_{s}^{Si}$ versus $n_{m}$, both $r_{LNP}$ and $n_{LNP}$ can be extracted by fitting the data to Eq. **3** (Fig. 1*B*), enabling multiparametric analysis of key properties of both high- and low-DSPC LNPs to be scrutinized and correlated. In our calculations, we define a reference scattering value $I_{s}^{Si}\left(n_{m}\right)$ as the median value of the scattering intensity from all surface-adsorbed silica NPs in the field of view (between 100 and 200 NPs in each measurement at a given medium refractive index and wavelength). Since theory predicts that the scattering intensity as a function of nanoparticle size will have local maxima and thus will decrease in certain size intervals, particles with a radius of above 115 nm are disregarded to avoid unreliable fits (SI Appendix, section 5 and Fig. S4). Fits with an $R^{2}<0.9$ are disregarded as outliers. It is also worth noting that while the more rigorous and broadly applicable Mie theory could have been implemented instead of the RGD approximation, the latter allows for experimental data to be fitted with closed-form analytical expressions and is therefore preferred in our analysis. Yet, to verify the eligibility of our approach, the two theories were compared within the examined range of refractive index difference and particle size regime demonstrating differences below 2.5%, which translates to a maximum difference of ~2 nm in the determination of radii and approximately 0.003 in the determination of the refractive indices (SI Appendix, section 6 and Tables S5 and S6). The particle scattering intensity calculated using Mie and RGD theory are also plotted as a function of particle radius and compared in SI Appendix, Fig. S5. To investigate how measurement noise affects the extracted particle size and refractive index values, an analysis based on simulated data was conducted, the results of which can be found in SI Appendix, section 7.

**Fig. 2.**
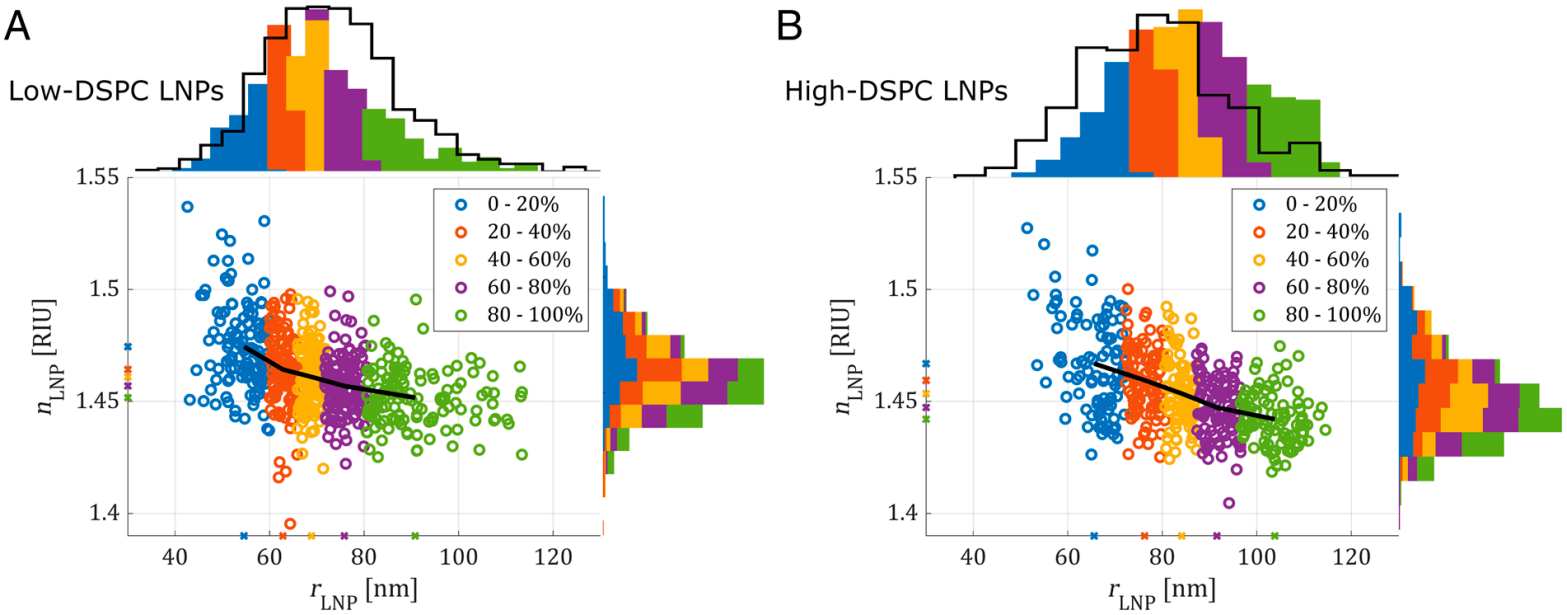
Scatter plots of $n_{LNP}$ versus $r_{LNP}$ for individual (*A*) low-DSPC LNPs and (*B*) high-DSPC LNPs with histograms projected onto the respective axes. The black curves on the radius histograms (*Top*) show the corresponding distribution from NTA measurements. The data are divided into five equally populated color-coded groups based on size. The black curve in the scatter plot indicates the mean $n_{LNP}$ and $r_{LNP}$ values for each size group, also indicated with marks on the figure axes.

Extraction of both radius and refractive index for individual LNPs is illustrated in scatter plots of $r_{LNP}$ versus $n_{LNP}$ for low-DSPC (Fig. 2*A*) and high-DSPC (Fig. 2*B*) LNP batches with the corresponding histograms projected on the respective axes. The size distributions (which display median radii values of 67 ± 2 nm and 80 ± 3 nm for the low-DSPC and high-DSPC LNPs, respectively) are in good agreement with those obtained using NTA of the same LNP batches (top histograms, black curves), although the NTA measurements give distributions that are slightly shifted to higher and lower size values for the low-DSPC LNPs and high-DSPC LNPs, respectively. A possible reason for this discrepancy is the higher amount of surface-exposed PEG at the surface of low-DSPC LNPs, resulting in a larger hydrodynamic radius measured by NTA, which is different from the optical radius obtained via the scattering signal (47). It is also worth noting that the low-DSPC LNPs have a radius that on average is 13 nm smaller and a size distribution that is 20% more narrow than that of the high-DSPC LNPs (Fig. 2 *A* and *B*, top histograms and SI Appendix, section 8 and Fig. S6).

In good agreement with previously obtained theoretical estimations based on an LNP water content of ~25% (12), the median refractive index estimated from the entire size distribution is ~1.462 ± 0.017 and ~1.455 ± 0.029 for the low-DSPC and high-DSPC LNPs batches, respectively (Fig. 2 *A* and *B*, side histograms). Moreover, inspection of the average refractive index value for each color-coded size regime (black curves in Fig. 2 *A* and *B*), smaller LNPs appear to exhibit a slightly higher refractive index than the larger ones, decreasing from ∼1.48 to ∼1.44 with increasing size. However, due to the inherent negative correlation between optical size and refractive index in this type of data analysis (i.e., underestimating one parameter leads to overestimating the other), careful consideration of the combined multiparametric data is required to determine the reliability of such trends. In fact, careful inspection of the raw data (SI Appendix, section 9) suggests that only high-DSPC LNPs display a moderate reduction in refractive index, from ∼1.46 to ∼1.44 with size, while the additional spread in estimated refractive index is attributed to experimental uncertainty.

To further elucidate these observations, it is instructive to investigate how the different LNP constituents are distributed within and among individual LNPs by inspecting how the fluorescence intensities of the Cy5-labeled mRNA cargo and Rhodamine-labeled DOPE-lipids vary across both LNP size and refractive index. The fluorescence intensity of an LNP ($I_{f}$) can be expressed in analogy with the above-treated scattering intensity (SI Appendix, section 4) (45).

**Fig. 3.**
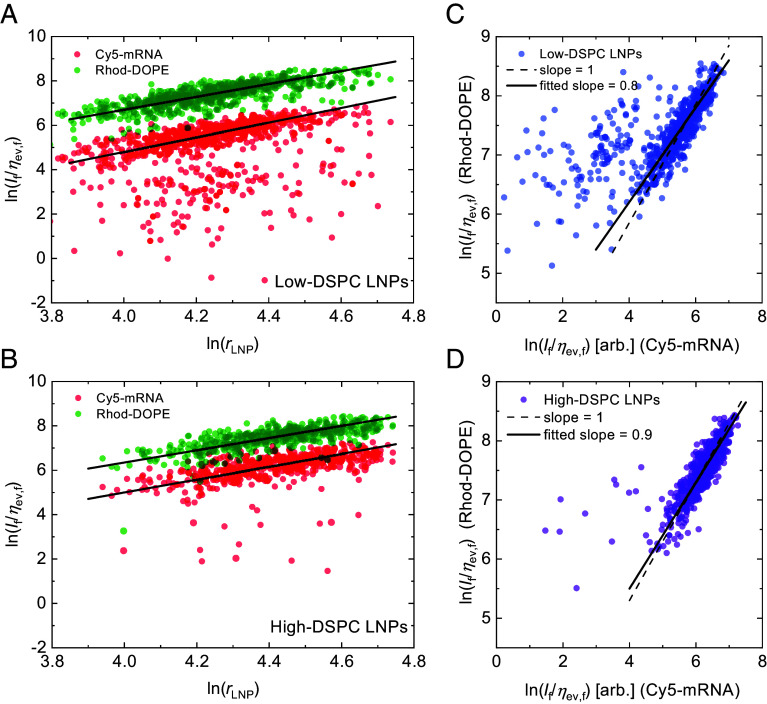
Log–log scatter plots of fluorescence intensities ($I_{f}/\eta_{ev,f}$) versus LNP radius ($r_{LNP}$) for Cy5-mRNA (semitransparent red dots) Rhod-DOPE (semitransparent green dots) for (*A*) low- and (*B*) high-DSPC LNPs, with regression lines fitted through the datasets neglecting outliers. The fitted lines through the low-DSPC LNPs in (*A*) have slopes of 2.9 and 3.3 for the Rhod-DOPE and Cy5-mRNA, respectively, and the corresponding slopes for the high-DSPE LNPs in (*B*) are 2.9 and 2.8, respectively. Log–log scatter plots of Rhod-DOPE versus Cy5-mRNA fluorescence intensities for (*C*) low- and (*D*) high-DSPC LNPs, respectively, with solid black lines representing fitted lines through the datasets and the dashed lines (with fixed slopes of 1), depicting the anticipated slopes in the case the two fluorescent components (Rhod-DOPE and Cy5-mRNA) have identical spatial distribution within the LNPs.

A log–log representation of the fluorescence emission intensities $\left(I_{f}/\eta_{ev,f}\right)$ versus $r_{LNP}$ for the low- and high-DSPC LNP formulations display similar power–law relationships with regression lines through the data having slopes close to 3 for both the Cy5-mRNA (Fig. 3*A*) and Rhod-DOPE (Fig. 3*B*) intensities. This power–law representation of the data is informative as it reveals details about the spatial distribution of the fluorescent material within the LNPs. If, for example, Rhod-DOPE lipids were colocalized with lipids near the surface of the LNPs, a slope of approximately 2 would have been anticipated in Fig. 3*B* ${(I}_{f}\propto r^{2}$), while the observed slope of 3 indicates a more homogeneous distribution of the labeled lipids throughout the particle volume ${(I}_{f}\propto r^{3}$). The former power-relationship of 2 between surface and volume distributed fluorescence was indeed confirmed in a different set of experiments conducted using LNPs containing Atto-488 labeled DMPE-PEG lipids (Atto-488-PEG), which are expected to reside on the outer surface of the LNP (SI Appendix, section 10 and Fig. S9). The measured slopes of around 3 for both Rhod-DOPE and Cy5-mRNA (Fig. 3 *A* and *B*), and a slope close to unity when plotted against each other (Fig. 3 *C* and *D*), thus suggest that both materials are distributed throughout the volume of the LNPs. Further, the slightly steeper slope of 3.3 for the Cy5-mRNA of the low-DSPC LNPs (Fig. 3*A*) and the slight deviation from unity of the fitted slopes in Fig. 3 *C* and *D*, is possibly an indication that for those LNPs, the mRNA material is concentrated in an inner core, surrounded by a thin Cy5-mRNA-free shell that does not vary in thickness across LNP size, presumably composed of DSPC and Rhod-DOPE lipids and in particular DMPE-PEG lipids with PEG polymers in a brush-like configuration (SI Appendix, section 11 and Fig. S10).

This interpretation is consistent with previously reported results for similar types of LNPs, from which it was concluded that DSPC lipids are colocalized with cholesterol and PEG-lipids at the LNP surface, while the mRNA-cargo is preferentially localized together with DLin-MC3-DMA and cholesterol in the LNP core (12, 48, 49). Furthermore, our observation that Rhod-DOPE is distributed within the entire volume of the LNPs, and not limited to the surface, as was the case for the Atto-488-PEG LNPs (SI Appendix, section 10 and Fig. S9), is attributed to the rhodamine label carrying a net negative charge which, in analogy with mRNA, is likely to be electrostatically attracted to DLin-MC3-DMA in the acidic conditions used during LNP fabrication. This result differs from that obtained in a separate but similar study where fluorescence intensity from the lipophilic membrane dye (Dil-C18) was observed to scale with the LNP surface area rather than volume (33, 34), highlighting the relevance of this type of analysis not only from a fundamental perspective but also to correctly interpret data when labels of this type are used to estimate LNP size distributions and to correctly interpret the fate of LNPs in in vitro cellular assays (32, 50).

**Fig. 4.**
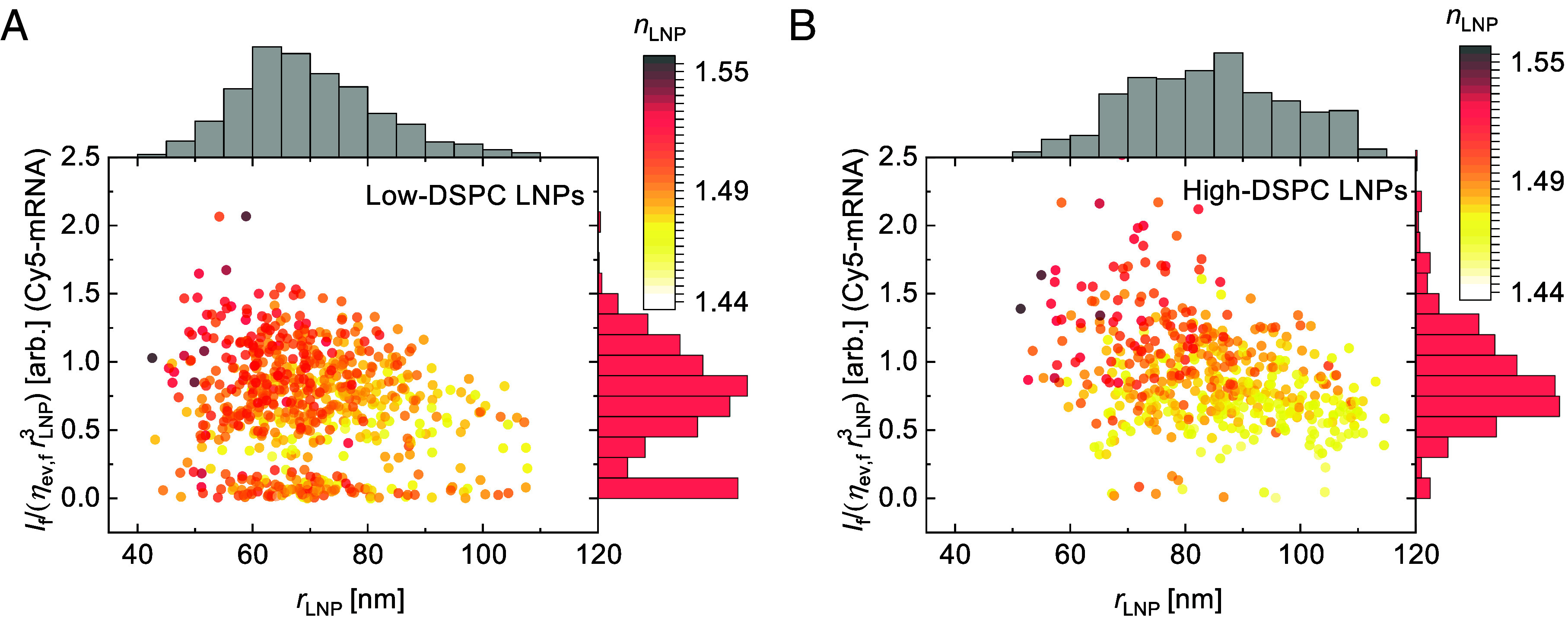
Scatter plot representation of $I_{f}/{(\eta }_{ev,f}r_{LNP}^{3})$ versus $r_{LNP}$ for individual LNPs as given in Fig. 2 for the (*A*) low-DSPC LNPs and (*B*) high-DSPC LNPs, here color-coded according to $n_{LNP}$.

Another notable feature in Fig. 3, observed for both types of LNP formulations, is the presence of LNPs with weak Cy5-mRNA fluorescence intensities, likely corresponding to particles lacking mRNA cargo (51). This subpopulation accounts for maximum 10% of the total number of LNPs in our samples. To analyze this observation further, the Cy5-mRNA fluorescence intensity per unit volume ($r_{LNP}^{3}$) was plotted versus LNP radius and color-coded according to the refractive index of the LNPs (Fig. 4). When represented this way, it becomes evident that the low-Cy5-mRNA-fluorescent subpopulation is distributed evenly across the entire LNP size regime, with a refractive index distribution that is similar to that of Cy5-mRNA-positive LNPs (see also SI Appendix, section 12 and Fig. S11 *A* and B), while a positive correlation is observed between refractive index and Cy5-mRNA fluorescence (per unit volume). The subpopulation displaying diminishing Cy5-mRNA fluorescence also displays a somewhat lower Rhod-DOPE fluorescence intensity than the average LNP (SI Appendix, section 12 and Fig. S11 *C* and D). The observed refractive index distributions observed in these populations are likely due to a variation in water content, rather than mRNA content (SI Appendix, section 13) (12, 49, 52).

Considering the impact of the uncertainty in the fluorescence emission signal on the analysis, the fluorescence intensities from spherical particles with identical size distributions as those used in our measurement were simulated, taking into account uncorrelated uncertainties in fluorescence intensities, refractive index values, and size estimation (SI Appendix, section 12 and Fig. S14). In analogy with the analysis of the refractive index variation across LNP size (Fig. 2), the simulations consistently indicate that the subtle negative correlation observed between fluorescence content and LNP size observed in Fig. 4 (see also SI Appendix, section 12 and Fig. S12) is most likely related to a combination of uncertainties in size estimation and to effects on the correlations of a thin nonfluorescent layer surrounding the fluorescent core of the LNPs (12, 48).

From the above analysis, we conclude that there is a clear correlation between Cy5-mRNA content and LNP volume, while the refractive index distribution primarily depends on differences in LNP water content. It is clear, though, that no major differences are observed between the two types of LNPs, especially when taking into account the likely influence of the shell of PEG on LNP size (Fig. 2 and SI Appendix, section 11 and Fig. S10). Indeed, despite the richness of information contained in this in-depth multiparametric analysis, it is not possible to identify clear differences between the two types of LNPs that can explain their more than one order of magnitude difference in functional mRNA delivery in vitro (12).

It is in this context worthwhile to note that the compositional difference between low-DSPC- and high-DSPC LNPs is characterized by a variation in the relative proportions of gel-phase forming DSPC and DMPE-PEG lipids (Table 1), which combined account for less than 15% of the overall LNP mass. Together with the fact that these two lipids are expected to be predominantly located at the surface of the LNPs (12, 48), it is not surprising that observed differences in refractive index and cargo distribution between the two LNP formulations are minor. The distinct difference in functional response between the two formulations is more likely related to how the different surface properties of the LNPs influence the translocation of Dlin-MC3-DMA lipids to the surface of the LNP as the pH of the endosome is lowered, which in turn controls the nature of electrostatic interaction with the endosomal membrane. To investigate this hypothesis, in an approach inspired by a previous investigation using total internal reflection microscopy (27, 53), we extended the analysis of SLB-tethered LNPs by also probing changes in both scattering and fluorescence intensities induced upon microfluidic-assisted reduction in pH from 7.4 to 6.0, intended to mimic the acidification within early endosomes (54, 55).

**Table 1.** LNP batches used in the study

Composition (lipids, mol-%)	Low-DSPC LNPs	High-DSPC LNPs	Atto-488-PEG LNPs
DLin-MC3-DMA	53.47	50	53.47
DSPC	4.65	10	4.65
Chol	41.114	39.684	41.114
DSPE-PEG-biotin	0.006	0.006	0.006
DMPE-PEG	0.7	0.25	0.35
Rhod-DOPE	0.06	0.06	–
Atto-488-DMPE-PEG	–	–	0.350

Lipid compositions are listed for the formulations used in the study.

**Fig. 5.**
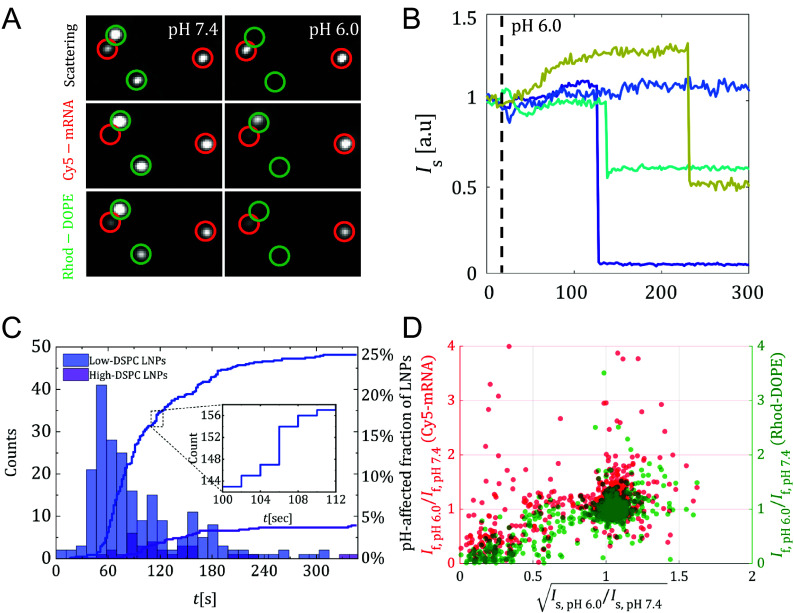
(*A*) Micrographs showing examples of four SLB-bound low-DSPC LNPs at the three recorded channels, scattering, Cy5-fluorescence, and Rhodamine fluorescence, before (*t* = 0 s) and after (*t* = 300 s) changing the buffer from pH from 7.4 to 6.0. Two of the LNPs exhibit an almost complete diminishing of the scattering intensity (green circles), accompanied by significant drops in both Cy5-mRNA and Rhod-DOPE fluorescence intensities, being attributed to LNP fusion with and transfer of lipid material to the underlying SLB, and escape of mRNA from the LNPs. In contrast, the other two display no significant changes in scattering intensity (red circles). (*B*) Examples of scattering intensity versus time for four low-DSPC LNPs that displayed a response upon the pH decrease. The dashed line indicates the time of the buffer exchange. (*C*) Solid curves show the cumulative number of particles displaying a decrease in scattering intensity following the pH decrease at *t* = 0 s for the low-DSPC LNPs (blue) and high-DSPC LNPs (purple). The *Left* y-axis shows the absolute number of responding particles while the *Right* y-axis shows these numbers normalized to the total amount of particles on the surface. These data are also presented in the delay-time (t) histograms at which the scattering intensity dropped. The histograms show data for all particles that responded to the pH decrease by a change in scattering intensity. For the low-DSPC LNPs, this fraction of LNPs displaying a sudden intensity drop varied from 10 to 25% between measurements, while the corresponding number for the high-DSPC LNPs was around 4 to 7%, even after prolonged (tens of minutes) exposure to pH 6.0 (SI Appendix, section 17 and Movies M2 and M3). (*D*) The relative change in fluorescence intensity versus the relative change in scattering intensity for individual low-DSPC LNPs, for Cy5-mRNA (red) and Rhod-DOPE (green). Particles with the 1% lowest initial Cy5 fluorescence intensity were excluded due to resulting high uncertainty in the intensity values.

Fig. 5*A* shows micrographs exemplifying time-resolved changes in the label-free scattering signal, as well as the Cy5-mRNA and Rhod-DOPE emission intensities for four representative SLB-bound LNPs prior to and after a reduction in pH from 7.4 to 6.0. Two of the LNPs exhibit an almost complete diminishing of the scattering intensity (green circles), accompanied by significant drops in both Cy5-mRNA and Rhod-DOPE fluorescence intensities. This feature is attributed to LNP fusion with and transfer of lipid material to the underlying SLB, and escape of mRNA from the LNPs. In contrast, the other two LNPs display no significant changes in scattering intensity (red circles). It should also be noted that if the LNPs are immobilized on the sensor surface using a binding strategy not involving a lipid membrane [using PLL-g-PEG-biotin (Poly(l-lysine)-graft-poly(ethylene glycol)) instead of the SLB] a reduction in the surrounding pH does not result in the stepwise particle intensity changes observed in the membrane-containing setup.

The heterogeneity in the response becomes even more evident from an inspection of the temporal evolution of scattering intensity, as illustrated for four additional particles upon reduction of the pH (Fig. 5*B*). Upon a pH drop at t∼20 s, three of the LNPs display a pronounced intensity decrease that varied from 40 to >90% after wait times which in this example ranged between 100 and 200 s, while one of the LNPs shows little or no response. Besides different wait times and magnitudes of the intensity drops, the LNPs display diverse behavior also prior to the sudden intensity drop. Upon a decrease in pH, two LNPs, depicted by the curves in navy blue and amber in Fig. 5*B*, initially display clear intensity increases, which is consistent with a translocation of the LNPs toward the surface where the intensity of the evanescent field is enhanced. This translocation is attributed to cationization of the LNPs induced by the pH decrease, thereby enhancing electrostatic attraction to the slightly negatively charged SLB-streptavidin substrate. It is also worth noting that a comparison between the low-DSPC LNPs that undergo fusion with those that do not, there is no clear correlation between particle size, refractive index, and fusogenicity (SI Appendix, section 15 and Fig. S16).

Taking all inspected LNPs into account, the distribution of the wait times for low- and high-DSPC LNPs display mean values of around 55 and 90 s, respectively, while the corresponding fraction of LNPs displaying sudden drops in scattering intensity of at least 40% were around 25 and 4%, respectively (Fig. 5*C*). The stark contrast between the two types of LNPs to undergo pH-induced fusion is attributed to the higher surface coverage of the gel-phase-forming DSPC lipid in high-DSPC LNPs and longer delay-times signals that a likely explanation to the difference is that DSPC hampers exposure of cationized DLin-MC3-DMA at the LNP surface. The pronounced difference in fusogenicity between the two LNP compositions is reflected in complementary experiments using primary human adipocytes and iPSC-derived human hepatocytes. Although both LNP types exhibit similar levels of cellular uptake, the low-DSPC LNPs lead to significantly higher expression of human erythropoietin (hEPO), attributed to a more efficient endosomal mRNA escape (SI Appendix, section 14).

When analyzing the low-DSPC LNP measurements and comparing the particles which undergo fusion with those that do not it is interesting to note that there does not seem to be any correlation between particle size, refractive index, and fusogenicity (SI Appendix, section 15 and Fig. S16).

Additional information can be gained by correlating changes in the scattering signal with the magnitude of the changes observed in the fluorescence channels. This is illustrated in Fig. 5*D*, which presents a scatter plot of ($I_{f}^{pH6.0}/I_{f}^{pH7.4}$) for both Cy5-mRNA and Rhod-DOPE versus (the square root of) the corresponding ratio in scattering intensity ($I_{s}^{pH6.0}/I_{s}^{pH7.4}$) before and after the pH reduction for all inspected low-DSPC LNPs. The scatter plot exhibits three distinct LNP populations: i) one which remains relatively unaffected by the pH decrease and clusters around the value 1 for all three axes, ii) one which experiences a rapid decrease in scattering intensity while maintaining a preserved fluorescence intensities, and iii) one which exhibits both a rapid reduction in scattering intensity and altered fluorescence intensities.

Out of the LNPs exhibiting a rapid decrease in scattering intensity (subpopulations ii and iii), a majority (∼72%) also displayed a significant (∼80%) reduction in Rhod-DOPE fluorescence intensity. This is indicative of lipid escape to the SLB, signaling a strong correlation between reductions in scattering and Rhod-DOPE emission intensities (green data points in Fig. 5*D*), being attributed to either structural changes (41) or more likely, mass loss of the LNP, which here manifests in lipid transfer from the LNP to the SLB, presumably induced by an electrostatic attraction between the cationic LNP and the (weakly) anionic SLB (56). The corresponding change in Cy5-mRNA fluorescence intensity for both subpopulation ii and iii was of lower magnitude and while in most cases constituting an intensity decrease, a few LNPs displayed an increase in intensity.

From the relative changes in Cy5-mRNA fluorescence emission, it is clear that the correlation with changes in scattering intensity is weaker than that observed for Rhod-DOPE, and although a reduction in Cy5-mRNA emission is observed for a majority of the LNPs displaying a drop in scattering intensity, an appreciable number of LNPs increase in Cy5-fluorescence emission by up to a factor of 1.5 and even above (Fig. 5*D*). The increase in Cy5-emission is consistent with previous TIRF microscopy measurements of LNP binding to planar endosomal membrane mimics, suggesting that upon LNP collapse at the membrane, mRNA is brought closer to the interface where the excitation intensity is enhanced (14). In contrast, the events in which a reduction in Cy5-mRNA emission was observed can be attributed to Cy5-mRNA-escape into solution and/or transfer into surrounding SLB, presumably aided by ionized DLin-MC3-DMA lipids electrostatically bound to mRNA (27).

It is also worthwhile to note that despite this significant heterogeneity in the fusion behavior, no clear correlations are observed between the three subpopulations and the measured particle size, refractive index, and Cy5-mRNA content (SI Appendix, Fig. S11). This suggests that characterization of these physicochemical properties alone is insufficient to fully capture the properties of mRNA LNP, as here demonstrated by the correlation between fusion efficiency in an in situ model (Fig. 5*B*) and cellular data for the two LNP types examined.

## Conclusions

By extending quantitative characterization of individual mRNA-containing LNPs beyond size and fluorescent cargo correlations (32), we have in this work presented an approach that offers quantitative mapping of how both size and refractive index of individual LNPs correlate with fluorescent cargo and lipid distribution. In comparison with previously reported means to determine the size of biological nanoparticles via their diffusion, enabling refractive index determination from the magnitude of the scattering signal (33, 34), size and refractive index of individual LNPs were in this work deconvoluted by probing scattering intensity variations induced by changing the refractive index of the medium surrounding SLB-tethered LNPs. This way of providing multiparametric characterization of individual LNPs is also directly compatible with time-resolved measurements of LNP dynamics, here demonstrated by combining the characterization with surface-based functional assays to investigate fusogenicity (27).

It is important to note, though, that a change in the surrounding medium may influence the structure of the LNPs, as for example reported upon exposure of DNA containing lipoplexes to polyethylene glycol (PEG) (57). Although the osmotic change induced in the concentration range of iodixanol used here is small (SI Appendix, section 3), one cannot completely exclude an influence on LNP structure. However, the $I_{s}^{LNP}/I_{s}^{si}$ ratio, from which size and refractive index were extracted, is fully reversible (SI Appendix, section 16 and Fig. S17), which indicates that any potential influence arising from alterations in iodixanol solution properties does not seem to lead to irreversible changes in the LNPs. Further, under the assumption that the LNPs can be considered as homogeneous spheres with an impermeable outer membrane, the mean refractive index of both low-DSPC- and high-DSPC LNPs was determined to ∼1.46, which is close to expected value if ~25% of the internal structure is composed of water (12), in agreement with LNPs being impermeable to iodixanol.

Our results are also consistent with previously predicted core-shell structure of the LNPs (Fig. 3), with mRNA confined to a core and a thin outer leaflet consisting mostly of cholesterol, DSPC, and PEG lipids in a bilayer configuration (12, 49, 52). Both the high- and low-DSPC LNPs contained a subpopulation (between 0 to 10% of the total population) exhibiting diminishing Cy5-mRNA fluorescence intensities, which we associate with LNPs deprived of mRNA. Empty LNPs are commonly observed within batches of small LNPs (<80 nm in diameter) that contain both few and relatively large mRNA molecules (36, 51). In our case, however, a typical LNP is around 120 nm in diameter is expected to contain on the order of hundreds of mRNA molecules (12). The fraction of empty LNPs varied extensively within and between LNP-batches, but they all exhibited a relatively broad variation in refractive index (Fig. 4 and SI Appendix, section 12 and Fig. S11). This is tentatively attributed to a variation in water content, although one cannot exclude that LNPs displaying high refractive index are permeable to iodixanol. Indeed, the existence of LNPs with diminishing Cy5-mRNA emission is indicative of mRNA escape from LNPs with compromised integrity, which could facilitate mRNA leaking during handling. However, if mRNA escape occurs at a similar rate from all LNPs, one would expect a broadening of Cy5-mRNA fluorescence intensity distribution rather than the distinct appearance of a subpopulation, adding yet one evidence to the broad heterogeneity of mRNA containing LNPs.

The observed heterogeneity with respect to size, refractive index, and cargo content was very similar for both low-DSPC LNPs and high-DSPC LNPs, and the minor differences observed (slightly larger mean size and broader size and refractive-index distributions for high-DSPC LNPs compared to low-DSPC LNPs) are not sufficient to explain the large difference in the pharmaceutical efficacy of these two LNP formulations, which suggests that the current intense endeavor to gain in-depth information about the compositional and structural variations of LNPs in particular, and biological nanoparticles in general, benefit from a possibility to connect quantitative physicochemical heterogeneity analysis with specific functions. This was here accomplished by investigating how a reduction in pH, mimicking the environment in early endosomes, influences the fate of LNPs when tethered to a planar-SLB. The results show that a reduction in the pH surrounding SLB-tethered low-DSPC LNPs induces the type of structural changes and material transfer events that are expected to be required for functional delivery of mRNA from the endosome to the cytosol, likely due to their lower surface coverage of gel-phase forming DSPC than that of high-DSPC LNPs, for which almost one order of magnitude fewer LNPs underwent similar changes (Fig. 5*C*). Yet, no clear correlations were observed between LNPs affected by the pH change (Fig. 5*D*) and their corresponding size, refractive index, or fluorescence characteristics prior to onset of fusion (SI Appendix, section 12 and Fig. S11) suggesting that particle characteristics, such as mRNA-loading efficiency, labeling protocols, refractive index or size, do not serve as the primary parameters responsible for pH-controlled interactions occurring between LNPs and the underlying SLB.

In summary, our results suggest that the factors that determine the efficiency by which LNPs can undergo fusion with a lipid membrane, a process believed to control the critical endosomal mRNA escape process required for cellular protein synthesis, cannot be easily judged even using an unprecedently detailed characterization of individual LNPs with respect to size, refractive index, and cargo content. However, utilizing surface sensitive microscopy in combination with SLB, serving as a simplistic mimic of a cell membrane, adds one additional dimension to the analysis of LNP function, here illustrated by the identification of stark difference between two LNPs with similar relatively similar lipid composition but with known functional differences in cell culture. Thus, by adding this dimension of particle characterization to the toolbox, the two LNP compositions could be distinguished in a way that correlates with their difference in biological functionality, underscoring the importance of moving beyond static LNP parameter characterization and adopting approaches that assess functional performance.

Although our liquid handling system enables rapid and robust liquid exchange (<5 s) with low sample volumes (<50 µL), the throughput of the presented multiparametric measurements is constrained by the necessity of using reference particles and different refractive index media to deduce the desired parameters. Background scattering from surface defects and/or adsorbed stray particles also impacts the maximum nanoparticle coverage to around ~1,500 per field of view, and also compromises the possibility to quantify nanoparticles below a radius of around ~15 nm. Nevertheless, there are no fundamental barriers to overcoming these limitations using chip designs with multiple fluidic channels and advanced liquid handling solutions similar to those employed in state-of-the-art surface plasmon resonance systems, including sophisticated optical solutions to increase the information content in the scattering signal.

Although beyond the scope of this work, it is worth noting that the approach devised in this work is also fully compatible with other types of SLBs that better mimic the true nature of the endosome. Waveguide-based label-free scattering microscopy also offer time-resolved inspection of biomolecular binding kinetics to individual biological nanoparticles (38, 40), thus adding yet another dimension which cannot be easily accomplished for suspended nanoparticles, but still being highly important in order to gain insights regarding for example protein corona formation (58) and other structural changes induced upon changes of the media surrounding LNPs prior to or after cellular uptake. Combined with alternative surface-based label-free methods that provide single nanoparticle resolution, such as optical interferometric methods like iSCAT (59), and atomic force microscopy for insights into the nanomechanical properties of soft biological nanoparticles (60), this approach could guide super-resolution microscopy investigations, which are presently undertaken using markers for specific endosomal types, (55) and LNP-induced membrane damage (44), ultimately aiming to pinpoint the mechanisms underpinning functional mRNA escape.

## Materials and Methods

### Preparation of Vesicles for SLB Formation.

Biotinylated vesicles were made of 95 mol-% 1-palmitoyl-2-oleoyl-glycero-3-phosphocholine (POPC) and 5 mol-% 1,2-distearoyl-sn-glycero-3-phosphoethanolamine-N-[biotinyl(polyethylene glycol)-2000] (DSPE-PEG(2000)-biotin) lipids purchased from Avanti Polar lipids. Lipid vesicles were produced by forming a lipid film in a round-bottom flask from lipids dissolved in chloroform by drying in a rotary evaporator and subsequent further vacuum-drying for 8 h. The film was rehydrated through the addition of phosphate-buffered saline (PBS) solution (137 mM NaCl, pH 7.4, Sigma-Aldrich) in quantities yielding a 1 mg/ml lipid concentration. The vesicles were subjected to five cycles of freeze-thawing using liquid nitrogen and a 40 °C water bath and subsequent extrusion through a polycarbonate membrane (100 nm pores, Whatman) 31 times.

### LNPs.

LNPs were prepared using a NanoAssemblr (Precision NanoSystems Inc.) for microfluidic mixing of lipids and RNA as described by Zhigaltsev et al. (25). The lipids were dissolved in ethanol at indicated molar ratios to a total lipid concentration of 12.5 mM (1.85 mg/mL). mRNA was diluted in RNase-free citrate buffer (50 mM, pH 3.0). The two solutions were mixed in a 3:1 volume ratio at a rate of 12 mL/min to obtain LNPs with a mRNA:lipid weight ratio of 1:10 (DLin-MC3-DMA:nucleotide; 3:1 molar ratio). The resulting LNPs were dialyzed overnight against sterile PBS pH 7.4 using 10 kDa cut-off Slide-A-Lyzer G2 dialysis cassettes (Thermo Scientific). Each batch of LNPs was characterized prior to use to determine particle size, polydispersity, particle concentration, and RNA encapsulation.

The ionizable cationic lipid O-(Z,Z,Z,Z-heptatriaconta-6,9,26,29-tetraem-19-yl)-4-(N,N-dimethylamino)butanoate (DLin-MC3-DMA) was synthesized by AstraZeneca. 1,2-distearoyl-sn-glycero-3-phosphocholine (DSPC),1,2-distearoyl-sn-glycero-3-phosphoethanolamine-N-[biotinyl(polyethylene glycol)-2000] (DSPE-PEG-biotin) and 1,2-dioleoyl-sn-glycero-3-phosphoethanolamine-N-(lissamine rhodamine B sulfonyl) (Rhod-DOPE) lipids were from Avanti Polar Lipids, 1,2-dimyristoyl-sn-glycero-3-phosphoethanolamine-N-[methoxy(polyethyleneglycol)-2000] (DMPE-PEG) were from NOF Corporation, and cholesterol from Sigma-Aldrich. CleanCap™ Cy5 eGFP mRNA (5-methoxyuridine) and CleanCap™ eGFP mRNA (5-methoxyuridine) (996 nucleotides) were from TriLink Biotechnologies. Citrate buffer (Teknova) and ethanol 99.5% were used in the LNP production; the mRNA was dissolved in HyClone HyPure Molecular Biology Grade RNase-free water (GE Healthcare). PBS (10X, pH 7.4) for dialysis, PBS tablets (−Ca2+, −Mg2+), the Quant-iT RiboGreen RNA assay kit, and Hoechst 33342 were purchased from Life Technologies.

### Waveguide Microscopy Measurements.

The waveguide chips with 450 nm spin-on-glass (SOG) core layer were provided by Nanolyze™. The chips were manufactured as described elsewhere (61) with an added deep reactive-ion etching processing step for making inlet and outlet holes through the structure to enable the efficient and rapid fluid exchange. Prior to use, the surface of the waveguide chip was rinsed in ultrapure H_2_O, isopropanol and dried in N_2_ and O_2_ plasma treated (30 W, Harrick Plasma cleaner) for 5 min. A flow cell on the chip was created by applying a 30 μm thick piece of double-sided black tape in which a channel had been formed using a laser cutter, on top of which a 170 μm thick glass slide was attached. The chip was placed in a Nanolyze™ Explore fluid handling and laser coupling system and light collection was achieved using an upright Olympus BX61 microscope equipped with a 60X, NA 1.0 water-immersion objective, a Hamamatsu ORCA-Flash 4.0 V2.0 CMOS camera, and a Hamamatsu W-VIEW GEMINI image splitter, containing specific filter cubes (SI Appendix, Tables S2) allowing for simultaneous acquisition of fluorescence and scattering signals. Illumination and fluorescence excitation were achieved by butt-coupling a TE-polarized single mode polarization-maintaining optical fiber to the end-facet of the waveguide chip. The other end of the optical fiber was connected to three different laser sources (SI Appendix, Table S3) using an optical fiber combiner (OZ Optics). Data were recorded at two frames per second, with camera exposure set to 50 ms.

After securing the chip in place, a continuous flow of PBS, at a rate of 10 μl/min, was initiated and subsequently, the injected solution underwent a sequential exchange at the same flow rate. First, a 0.1 mg/ml solution of biotinylated vesicles was injected until the formation of a SLB was observed visually [or 50 μg/mL Poly(l-lysine)-graft-poly(ethylene glycol)((PLL(20)-g (3.5)-PEG(2), SuSoS AG), of which 5% was functionalized with biotin for 15 min]. This was followed by a 40 µg/ml streptavidin solution, which was injected for 15 min, a 20 pM LNP solution, and finally a 150 nm diameter silica nanoparticle solution. The latter two steps were continued until a surface coverage of approximately 1,000 particles per field of view in total was achieved. Between each step, the chip was rinsed with PBS for 10 min to remove any excess material. Data of the LNP fluorescence and scattering intensity for all three wavelengths were then recorded after which the particle scattering intensity was recorded at 488 nm wavelength while the medium refractive index was sequentially varied by injecting Iodixanol (OptiPrep) solutions of 0, 5, 10, 15, 20 and 30 w/v-%, diluted in PBS ($n_{m}=1.333,1.342,1.349,1.360,1.372,1.387$). At each Iodixanol concentration, 100 frames were acquired at 50 ms camera exposure. All experiments were carried out at room temperature.

In order to minimize photobleaching of the samples, all measurements that did not directly rely on fluorescence readout were carried out using the 488 nm laser-line in scattering mode. This includes the series of image-acquisition in different iodixanol exchanges required for the evaluation of particle size and refractive index. Furthermore, even if some photobleaching cannot be totally ruled out, the scaling analysis presented in Fig. 3 is not affected by slight photobleaching since the slopes observed are determined by the relative fluorescence intensities within a single measurement rather than the absolute values.

Regarding other potential photophysical effects, it is important to note that each Cy5-labeled mRNA molecule, which constitutes 20% of the total mRNA cargo, contains approximately 34 Cy5 dyes. Given that an LNP with a 70 nm radius accommodates around 200 mRNA molecules (12) and given that the molecules are evenly distributed, the average distance between dyes is approximately 10 nm, which is significantly larger than the Cy5 Förster radius of 6 nm (62). Similarly, FRET interactions between the rhodamine and Cy5 labels are also likely to be small, but will nevertheless not affect the measured Rhodamine signal, as the long-pass filter used during 532 nm illumination transmits both emission signals.

### Data Extraction from Waveguide Microscopy Measurements.

Measurements conducted as described above, in which surface immobilized LNPs and silica nanoparticles immersed in a sequence of iodixanol solutions of different concentrations were observed, produced a stack of images which were processed as follows: The images representing scattering data were registered over time through a Fourier transform-based phase correlation method (63), after which the fluorescence images were aligned to their scattering counterparts, taking translation, rotation, scale, and shear transformations into account. The LNPs were identified and distinguished from background by locating local maxima in a fluorescence image while the silica particles were identified based on images recorded before and after the particle addition. To reduce effects of noise and background, the images were subsequently processed by Gaussian filtering and morphological image opening. To estimate the scattering and fluorescence intensity from individual LNPs, an area covering each identified particle and an associated local background area, free from particles, were selected, after which the pixel intensity values minus the average local background was integrated for every particle [see SI Appendix from Ref. 61 for more detailed description].

### LNP Uptake and hEPO Quantification.

Detailed descriptions of the procedures used for LNP cell uptake and expression measurements can be found in ref. 12.

## Supplementary Material

Appendix 01 (PDF)

Movie S1. Injection LNPs to streptavidin modified supporting lipid bilayer monitored using 488 nm light in scattering. The data was captured at a frame rate of 0.5 f/s (but the uploaded movie is sped up 50x), and field of view is approximately 100 μm.

Movie S2. Low-DSPC LNPs, bound to a streptavidin modified supported lipid bilayer, as the surrounding solution is exchanged from PBS at pH 7.4 to PBS at pH 6. The data was captured at a frame rate of 0.5 f/s (but the uploaded movie is sped up 50x), and field of view is approximately 100 μm.

Movie S3. High-DSPC LNPs, bound to a streptavidin modified supported lipid bilayer, as the surrounding solution is exchanged from PBS at pH 7.4 to PBS at pH 6. The data was captured at a frame rate of 0.5 f/s (but the uploaded movie is sped up 50x), and field of view is approximately 100 μm.

## Data Availability

Microscopy data and matlab code data have been deposited in Zenodo (https://doi.org/10.5281/zenodo.14530409) (64).
