# Supplementary material for: Multiparametric functional characterization of individual lipid nanoparticles using surface-sensitive light-scattering microscopy
Source: Proc Natl Acad Sci U S A. 2025 May 22;122(21):e2426601122. doi: 10.1073/pnas.2426601122 (PMC12130878; doi:10.1073/pnas.2426601122)
Supplement: Supplementary file 1 — Appendix 01 (PDF) [file pnas.2426601122.sapp.pdf]

PNAS

Supporting Information for
Multiparametric Quantification and Functional Characterization of Individual Lipid Nanoparticles Using Surface-Sensitive Light-Scattering Microscopy.

Mattias Sjöberg[a,b]*, Erik Olsén[a], Mokhtar Mapar[a], Petteri Parkkila[a], Simon Niederkofler[a], Sara Mohammadi[a], Yujia Jing[c], Gustav Emilsson[c], Lennart Lindfors[c], Björn Agnarsson[a]*, and Fredrik Höök[a]*

[a] Division of Nano and Biophysics, Department of Physics, Chalmers University of Technology, Gothenburg, Sweden.

[b] Nanolyze, Gothenburg, Sweden.

[c] Advanced Drug Delivery, Pharmaceutical Sciences, R&D, AstraZeneca, Gothenburg, Sweden.

Email: mattias.sjoberg@chalmers.se, *bjorn.agnarsson@chalmers.se*, *fredrik.hook@chalmers.se*

This PDF file includes:

Supporting text

1. Silica reference particles size distribution

The size distribution of the silica nanoparticles used as reference scatterers in the waveguide scattering microscopy measurements, as measured using Nanoparticle tracking analysis (NTA).

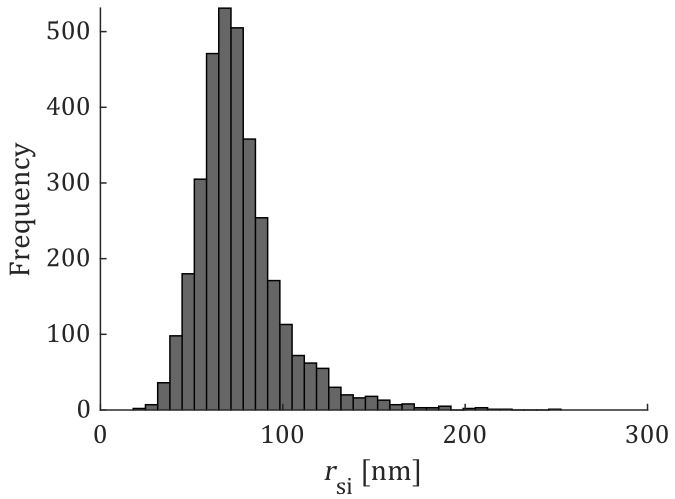

Fig. S1. NTA data showing size distribution for the silica nanoparticles used as reference particles in the waveguide scattering microscopy measurements. The mean radius of the distribution is $r_{Si} = 75$ nm.

2. Optical setup

Illustration of the experimental setup used for the waveguide scattering microscopy measurements, including the used laser, dichroic mirror, and optical filters.

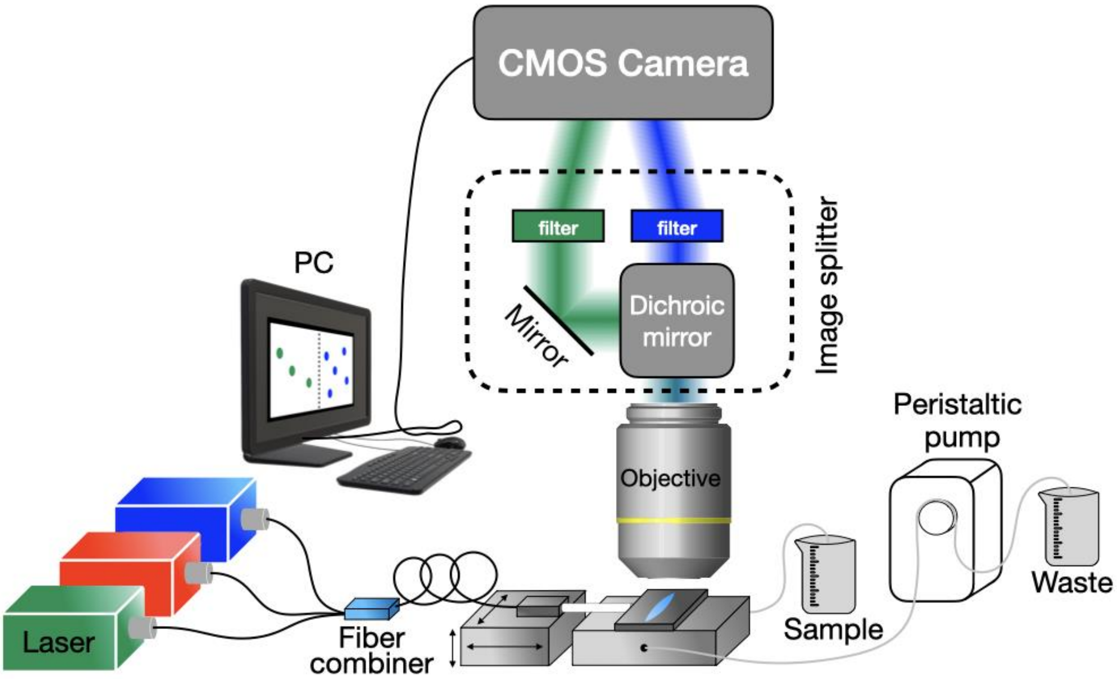

Fig. S2. Schematic illustration of the experimental configuration. Excitation light is provided by three linearly polarized lasers (Table S3) joined into a single mode optical fiber using an OZ Optics optical fiber combiner. The laser-light is coupled into the planar waveguide by aligning the fiber to the end-facet of the waveguide-chip using a *xyz*-translational stage. Scattering and fluorescence light from

surface bound particles interacting with the evanescent field of the bound waveguide-mode are collected by a microscope objective and projected onto an image splitter thus allowing for simultaneous monitoring of scattering and fluorescence intensities using appropriate filters (Table S2). Fluidic control and exchange are accomplished using a peristaltic pump connected to the outlet of the waveguide chip while the input is connected to a vial/beaker containing the sample.

Table S1. Wavelength parameters, expressed in nm, for the optical filters and dichroic mirrors used in the image splitter for acquiring the scattering and fluorescence signals. All filters are bandpass filters with properties specified as center wavelength/full bandwidth, except for the fluorescence filter for the 532 nm illumination, which is a long-pass filter.

**Illumination wavelength [nm]**	488	532	635
**Scattering filter (center/width)**	488/10	535/30	625/30
**Fluorescence filter (center/width)**	535/50	590 LP	700/75
**Dichroic mirror (center)**	510	580	660

Table S2. The lasers used in the experiments. Note that the OZ optics laser output is controlled using voltage. Operational power is here defined as the power setting of the laser, before fiber-coupling meaning that the actual powers coupled into the waveguide chips are considerably lower than the reported operational powers.

**Wavelength [nm]**	**Laser model**	**Operational power**
488	Cobolt, 06-01 series, CW, diode laser source	1 mW
532	NANO 250, QiOptiq, CW, diode-pumped solid state laser source	30 mW
635	OZ, 3000 series, CW, diode laser source	1866 mV

3. Optical and physical properties of iodixanol (OptiPrep^{TM})

OptiPrep^{TM} consists of 60 w/v% iodixanol in water. It has a density of 1.320 +/- 0.001 g/ml, endotoxin < 1.0 EU/ml and osmolality of 170 +/- 15 mOsm. All presented scattering data was collected using a wavelength of $\lambda_0 = 488$ nm. The refractive index values (RI) for the various iodixanol concentrations used in our measurements are based on an interpolation of refractive index values from literature, which presents iodixanol RI values at $\lambda_0 = 589.3[1]$ and $\lambda_0 = 589.3$ [2].

Table S3. Refractive indices of iodixanol for wavelengths $\lambda_0 = 589.3[1]$ and $\lambda_0 = 532$ nm[2], from which refractive index at $\lambda_0 = 488$ nm was evaluated using interpolation.

	**0 w/v%**	**5 w/v%**	**10 w/v%**	**15 w/v%**	**20 w/v%**	**30 w/v%**
$n_m(\lambda_0 = 589.3$ nm)	1.333	1.341	1.349	1.357	1.364	1.381
$n_m(\lambda_0 = 532$ nm)	1.333	1.342	1.349	1.359	1.369	1.384
$n_m(\lambda_0 = 488$ nm)	1.333	1.342	1.349	1.360	1.372	1.387

4. Nanoparticle intensity as a function of size and refractive index

For a particular wavelength, the measured inelastic light-scattering intensity ($\boldsymbol{I_s}$) of a surface-bound sub-wavelength particle in an evanescent illumination field can be expressed as[3]:

$$\boldsymbol{I_s = I_s^0 \eta_{ev,s} \eta_{RGD}}, \qquad (S1)$$

where

$$\boldsymbol{I_s^0 = A(\lambda_0, n_m)|\alpha|^2} \qquad (S2)$$

is the intensity calculated in the Rayleigh limit, $\boldsymbol{\alpha}$ is the polarizability of the particle and $\boldsymbol{A(\lambda_0, n_m)}$ is a function that includes the square of the illumination field intensity at the surface as well as the capturing efficiency of the optical setup, wavelength dependence and the sensitivity of the camera; while $\boldsymbol{\eta_{ev,s}}$ and $\boldsymbol{\eta_{RGD}}$ are dimensionless correction factors accounting for the exponentially decaying evanescent field and phase shifts according to the RGD approximation, respectively. By assuming that the surface-bound particles have a homogeneous spherical geometry of radius $\boldsymbol{r}$ the correction factors can be expressed as[3]

$$\boldsymbol{\eta_{ev,s} = 9\left(\frac{2\delta}{r}\right)^6 e^{-r/\delta}\left[\frac{r}{2\delta}\cosh\left(\frac{r}{2\delta}\right) - \sinh\left(\frac{r}{2\delta}\right)\right]^2}, \qquad (S3)$$

where

$$\boldsymbol{\delta = \frac{\lambda_0}{4\pi}\frac{1}{\sqrt{n_{eff}^2 - n_m^2}}} \qquad (S4)$$

is the penetration depth of the evanescent light intensity (with $\boldsymbol{n_{eff}}$ being the effective refractive index for the fundamental mode of the waveguide and $\boldsymbol{n_m}$ the refractive index of the surrounding medium), and

$$\boldsymbol{\eta_{RGD} = \left|\frac{3}{u^3}[\sin(u) - u\cos(u)]\right|^2, \; u = 2\left(\frac{2\pi n_m}{\lambda_0}\right) r \sin\left(\frac{\vartheta}{2}\right)} \qquad (S5)$$

where $\boldsymbol{\lambda_0}$ is the vacuum wavelength of the light and $\boldsymbol{\vartheta}$ is the angle between the incident and scattered light (which here is approximated to $\boldsymbol{\vartheta = \pi/2}$). The polarizability of a homogeneous dielectric sphere with refractive index $\boldsymbol{n}$ is given by[4]:

$$\boldsymbol{\alpha = \frac{4\pi r^3}{3}\frac{n^2 - n_m^2}{n^2 + 2n_m^2}} \,. \qquad (S6)$$

Parameters, $\boldsymbol{A(\lambda_0, n_m)}$ and $\boldsymbol{\delta(n_m)}$, introduced in Eqs. 2 and 3, respectively, are dependent on wavelength, light polarization and geometrical and optical properties of the materials defining the waveguide structure, which can both be evaluated by solving the corresponding Maxwell's equations using numerical methods[5] and be expressed as polynomial functions of $\boldsymbol{n_m}$ alone. In this way the measured I_s values for individual nanoparticles can be plotted versus n_m and the data fitted to Eq. 1 using r and n as free fitting parameters. However, a more practical way of obtaining parameters r and n is to compare a particle scattering intensity to that of a reference particle (I_s^{ref}) of known radius (r_{ref}), and refractive index (n_{ref}), and presenting the data as the ratio

$$\frac{I_s}{I_s^{ref}} = \frac{|\alpha(n)|^2}{|\alpha(n_{si})|^2}\frac{\eta_{ev,s}\,\eta_{RGD}}{\eta_{ev,s}^{ref}\eta_{RGD}^{ref}} \,. \qquad (S7)$$

In this way, the parameter $\boldsymbol{A}(\boldsymbol{\lambda_0}, \boldsymbol{n}_{\mathbf{m}})$ is eliminated, along with its inherent dependence on the surface field intensity as well as effects due to variations in the optical configuration and intrinsic intensity fluctuations in the illumination source. The remaining parameter, $\boldsymbol{\delta}(\boldsymbol{\lambda_0}, \boldsymbol{n}_{\mathbf{m}})$, can be expressed as a polynomial function of $\boldsymbol{n}_{\mathbf{m}}$ (see Fig. S3) and implemented in Eq. S3.

Penetration depth in different media

The extent of the evanescent field (penetration depth) into the cladding layer (medium) of the single-mode waveguide and field intensity at the surface of the core-cladding interface are dependent on the waveguide geometry, refractive indices and wavelength of the propagating light. In our measurements we apply three different wavelengths, λ_0; 488 nm, for acquiring scattering data and for fluorescence excitation of the Atto-488 fluorophore, 532 nm, for Rhod-DOPE fluorescence excitation and 635 nm, for Cy5-mRNA excitation. Furthermore, the scattering data is acquired in different media (n_{m}) by systematically changing between buffers containing varying proportions of iodixanol (see Table S1).

The penetration depth for each n_{m} and λ_0 can be easily obtained using Eq. 4 in the main text, where the effective refractive index of the propagating light is obtained using standard optical mode solver[5]. In our calculations we assume spin-on-glass (SOG) core layer thickness of 450 nm.

In Table S4 we summarize the calculated penetration depths used in our analysis.

Table S4. Penetration depths (δ) at different wavelengths according to Eq. S4 assuming SOG (waveguide core layer) thickness of 450 nm. Refractive indices of SOG and CYTOP (waveguide lower cladding layer) are obtained using an ellipsometer.

	$\boldsymbol{n}_{\mathbf{m}}$ $\mathbf{1.333}$	$\boldsymbol{n}_{\mathbf{m}}$ $\mathbf{1.342}$	$\boldsymbol{n}_{\mathbf{m}}$ $\mathbf{1.349}$	$\boldsymbol{n}_{\mathbf{m}}$ $\mathbf{1.360}$	$\boldsymbol{n}_{\mathbf{m}}$ $\mathbf{1.372}$	$\boldsymbol{n}_{\mathbf{m}}$ $\mathbf{1.387}$
$\lambda_0 = 488$ nm<br>$n_{\mathrm{SOG}}(488)$ =1.423<br>$n_{\mathrm{CYTOP}}(488)$ =1.338	99 nm	107 nm	114 nm	131 nm	157 nm	227 nm
$\lambda_0 = 532$ nm<br>$n_{\mathrm{SOG}}(532)$ =1.421<br>$n_{\mathrm{CYTOP}}(532)$ =1.337	113 nm	-	-	-	-	-
$\lambda_0 = 635$ nm<br>$n_{\mathrm{SOG}}(635)$ =1.417<br>$n_{\mathrm{CYTOP}}(635)$ =1.335	150 nm	-	-	-	-	-

In the main text, a curve fitting procedure is described where measured particle intensity as a function of n_{m}, in combination with known properties of the silica reference particles, $\left(I_{\mathrm{s}}/I_{\mathrm{s}}^{\mathrm{ref}}\right)|\alpha(n_{\mathrm{si}})|^2\eta_{\mathrm{ev,s}}^{\mathrm{ref}}\eta_{\mathrm{RGD}}^{\mathrm{ref}} = f(n_{\mathrm{m}})$ is used as data points. This data is fitted to Eq. 1 in the main text using r_{LNP} and n as free parameters, i.e.

$$f(n_{\mathrm{m}}) = |\alpha(n)|^2\eta_{\mathrm{ev,s}}\,\eta_{\mathrm{RGD}} \qquad \text{(S8)}$$

This curve fitting requires expressing the evanescent-light penetration-depth, δ, as a function of n_{m} for each wavelength used. $\delta(\lambda_0, n_{\mathrm{m}})$-values were calculated using Eq. S4 by first determining the effective refractive index (n_{eff}) of the propagating light for each n_{m} and wavelength λ_0 used in our experiments using an optical mode solver[5]. A 3rd degree polynomial function was then fitted to the curve and that function applied in Eq. 1 during fitting. An example of polynomial fitting to $\delta(488, n_{\mathrm{m}})$ is given in Fig. S3.

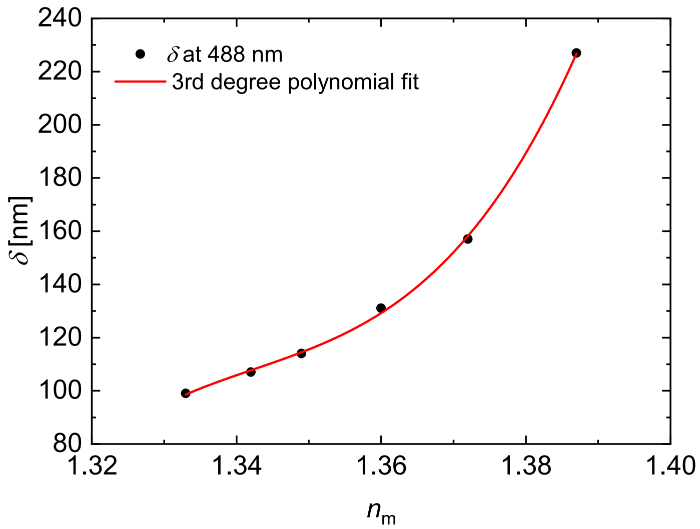

Fig. S3. A 3rd degree polynomial fit to $\delta(488, n_{\mathrm{m}})$ according to Eq. S4 using numerically obtained n_{eff}-values from optical mode solver[5].

Fluorescence intensity

In analogy to the scattering intensity discussed above (Eqs. S1-S5), the fluorescence intensity (I_{f}) of a particle illuminated by an evanescent field can in be expressed as [3]

$$I_{\mathrm{f}} = I_{\mathrm{f}}^{0}\eta_{\mathrm{ev,f}}, \qquad (S9)$$

where I_{f}^{0} is a function that includes the illumination field intensity at the surface, the capturing efficiency of the optical setup, wavelength dependence and the sensitivity of the camera, while $\eta_{\mathrm{ev,f}}$ is the dimensionless correction factor accounting for the exponentially decaying evanescent field, which in the case of fluorescence can be expressed as

$$\eta_{\mathrm{ev,f}} = 3\left(\frac{\delta}{r}\right)^{3} e^{-r/\delta}\left[\frac{r}{\delta}\cosh\left(\frac{r}{\delta}\right) - \sinh\left(\frac{r}{\delta}\right)\right]. \qquad (S10)$$

5. Exclusion of large particles due to uncertain particle parameter estimates

When there is an angle between the excitation light and collected scattered light, which in the waveguide experiments is approximately 90 degrees (neglecting the acceptance angle of the optics), the phase contribution to the scattering intensity of a nanoparticle will cause local maxima for certain particle radii, see (*A*). Since the intensity value will in this regime no longer uniquely correspond to a single particle size, fits based on the scattering intensity are unstable for particles of a size close to the size corresponding to such a maximum. For the waveguide setup used in this work, the first such maximum occurs at a radius around 120 nm. Based on the NTA data for the LNPs [black curves in top histograms in Figs. 2*A* and 2*B* in main text] only the tail of the distribution has a radius larger than 110 nm. Due to the poor fitting for particles with a radius larger than 110 nm, the majority of such particles will be assigned a radius of ~120 nm upon the fitting procedure described in the main text. To avoid influence of this instability during the size-correlation with for example fluorescence or refractive index, particles with an assigned radius from the fitting larger than 115 nm (red) are excluded during the analysis, see (*B*).

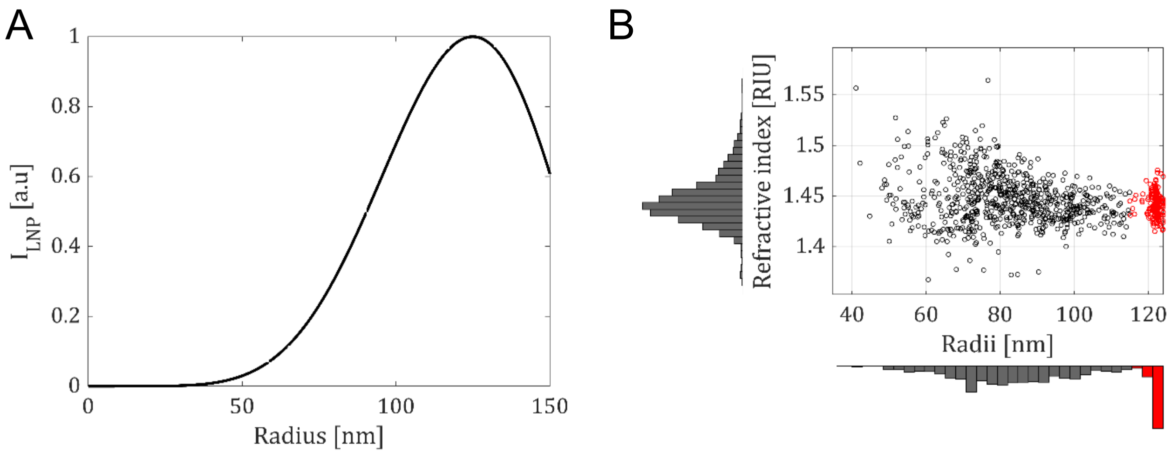

Fig. S4. The scattering intensity of a simulated LNP as a function of particle radius, showing that the phase contribution to the scattering intensity of a nanoparticle will cause local maxima for certain particle radii, see (*A*). Since this effect causes poor fitting of particles above 115 nm in radius (red circles in B), these are excluded from the analysis.

6. RGD vs. Mie

Tables S5 and S6 show the relative difference in r_{LNP} determined using the procedure shown in Fig. 2 in the main text for LNPs that produce similar scattering intensities when determined based on the Rayleigh-Debye-Gans (RGD) approximation and the complete Mie description.

Table S5. The relative difference in r_{LNP} determined using the procedure shown in Fig. 2 in the main text for LNPs that produce similar scattering intensities when determined based on the Rayleigh-Debye-Gans (RGD) approximation and the complete Mie description. The values are calculated for three different radius and refractive index values.

r_{LNP} [nm] / n_{LNP} [RIU]	40	75	110
1.42	0.5%	0.3%	2%
1.445	0.4%	0.05%	1.9%
1.47	0.2%	0.5%	2%

Table S6. The relative difference in n_{LNP} determined using the procedure shown in Fig. 2 in the main text for LNPs that produce similar scattering intensities when determined based on the Rayleigh-Debye-Gans (RGD) approximation and the complete Mie description. The values are calculated for three different radius and refractive index values.

r_{LNP} [nm] / n_{LNP} [RIU]	40	75	110
1.42	0.6%	1.4%	1.8%
1.445	0.7%	2.4%	1.0%
1.47	2%	2.1%	0.6%

Figure S5 shows a comparison between the Mie and RGD scattering intensity as a function of particle radius. The green dashed line shows the ratio between the two. As can be seen, for particles with radii below 120 nm, the difference between the Mie and RGD models is minimal, with the ratio remaining within 5%.

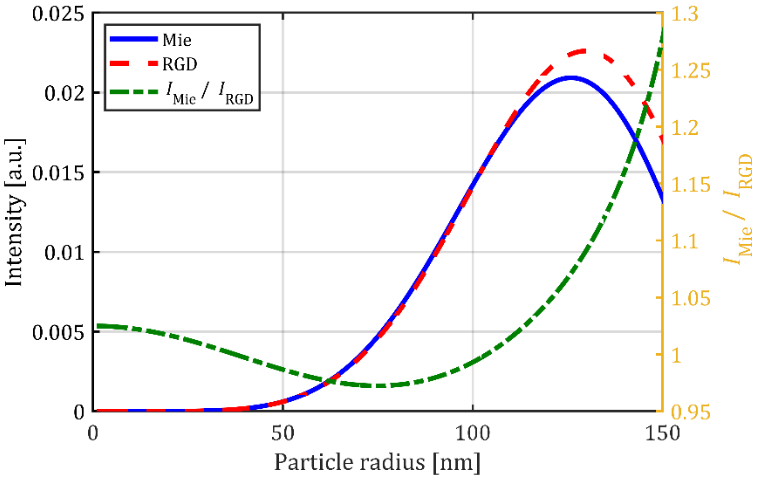

Fig. S5. The intensity of Mie (blue line) and RGD (red line) scattering as a function of particle radius (x-axis). The green dashed line represents the ratio of Mie to RGD scattering intensities (right y-axis). The data demonstrates that for particles with radii below 120 nm, the difference between the Mie and RGD models is minimal, with the ratio remaining within 5%. For larger particle sizes, the discrepancy between the two models increases. The intensity values are given in arbitrary units (a.u.). The plot is presented with a dual y-axis: the left axis for intensity and the right axis for the Mie-to-RGD ratio.

7. Size and refractive index uncertainty quantification

The measured LNPs exhibit intensity fluctuations due to noise, averaging 2.81% over time. To quantify measurement uncertainty and determine expected error margins, we introduce Gaussian noise at this level into the intensity of a simulated particle with a 70 nm radius and a refractive index of 1.45. The analysis program then retrieves a radius of 69.76 ± 1.14 nm and a refractive index of 1.4508 ± 0.0031.

8. Size distribution of LNPs

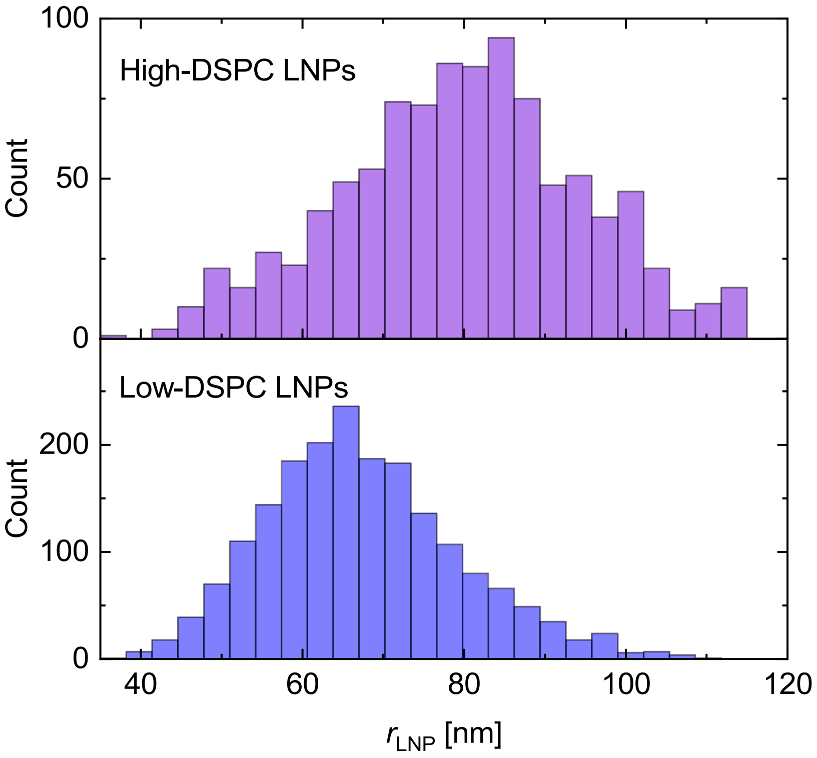

Fig. S6. Collective size distribution from four measurements of high- and low-DSPC LNP-batches obtained using the methodology described in the main text. The data presented in the top-histograms in Figs. 2*A* and 2*B* in the main text correspond to single measurements of low- and high-DSPC LNPs.

9. Refractive index distribution of LNPs

In Fig. 2 of the main text, there is an apparent LNP size-refractive index correlation, where the estimated refractive index decreases as the estimated particle size increases. However, since the measured intensity value of a particle is a function of the product of size and refractive index ($I_s \propto \alpha^2 \propto r^6 n^2$ - see Eqs. 2 and 6 of the main text), the estimated values of size and refractive index will affect the value of the other such that the corresponding scattering intensity remains the same. Thus, any uncertainty in the parameter estimations will therefore be inversely correlated, where an overestimation of one parameter will produce an underestimation of the other. Due to the presence of noise and optical aberrations during the measurements, where all such contributions will affect the particle scattering intensity, there is a risk of size-refractive index correlations in the data that are non-physical. Such correlations in the size-refractive index plot, although rarely commented, commonly occurs in the literature when for example measuring dielectric reference particles such as silica and polystyrene[6], [7], [8]. This spread of size and refractive index values are typically higher for particles close to the detection limit of the used method, which is the origin of the increased spread in values for the smallest detected particles in the Main text. Thus, such correlation in the size-refractive index estimation is not a unique feature of waveguide microscopy.

To assess if the observed size-refractive index correlations are physical or comes from uncertainty in the scattering intensity measurements, the ratios between the measured LNP and silica NP scattering intensities $I_\mathrm{s}^\mathrm{LNP}/I_\mathrm{s}^\mathrm{Si}$ are plotted versus n_m (Fig. S7 C and D, where the colors correspond to the color coding of the size intervals in Fig. S7A and B). Since the ratio between $\eta_\mathrm{ev.s}\eta_\mathrm{RGD}$ and $\eta_\mathrm{ev,s}^\mathrm{Si}\eta_\mathrm{RGD}^\mathrm{Si}$ is only weakly dependent on n_m for nanoparticles in the investigated size range, an inspection of Eqs. 6 and 7 in the main text reveals that the $I_\mathrm{s}^\mathrm{LNP}/I_\mathrm{s}^\mathrm{Si}$ ratio is expected to increase with increasing n_m for LNPs having higher n_LNP than that of the reference particles (n_Si ~ 1.45) and decrease if having a lower. This can be seen in Fig. S7 where Eq. 7 in the main text is used to produce theoretical curves showing this trend.

From Fig S7 it is clear that a negative trend implies that the LNPs have a refractive index below that of the silica reference particles ($n_\mathrm{Si} = 1.45$) and vice versa. A comparison between the slopes of the measured and theoretical curves reveals a refractive index similar to that of the reference particles for the low-DSPC LNP subpopulations while the high-DSPC LNP subpopulations show a weak trend of decreasing refractive index with increasing LNP size (with values ± 0.01 from the reference particle RI, i.e. 1.44-1.46). Additional spread thus likely comes from sources such as the presence of camera noise and scattering from neighboring particles and impurities inside the waveguide, where this uncertainty in the measured intensity value affects the spread of the size and refractive index estimations. Thus, the trend of a decreasing RI with increasing LNP size seen in fig 2A and B is likely a combination of a real effect and measurement and analysis uncertainties.

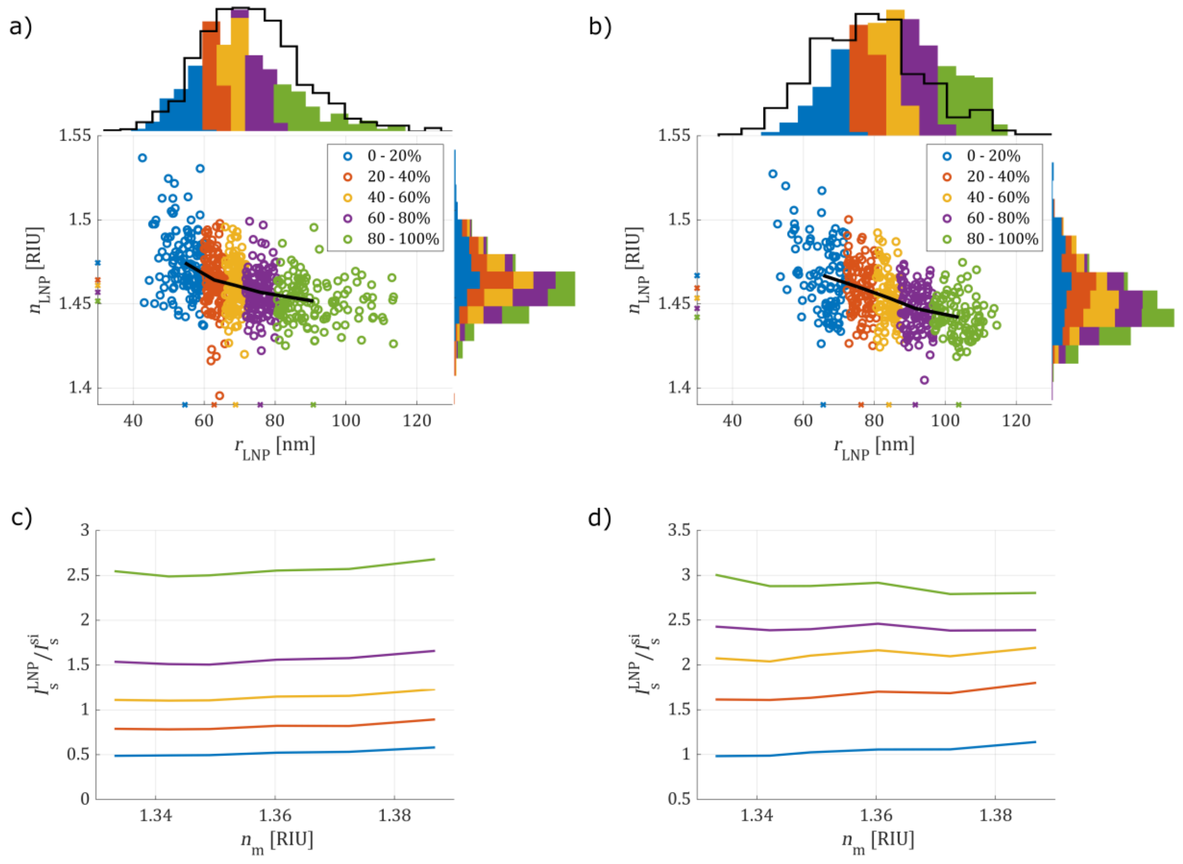

Figure S7. Scatter plots of n_{LNP} versus r_{LNP} for individual (*A*) low-DSPC LNPs and (*B*) high-DSPC LNPs with histograms projected onto the respective axes (Note that these are the same plots as in Fig 2 of the main text). The black curves on the radius histograms (top) show the corresponding distribution from NTA measurements. The data is divided into five equally populated color-coded groups based on size. The black curve in the scatter plot indicates the mean n_{LNP} and r_{LNP} values for each size group, also indicated with marks on the figure axes. (*C*) and (*D*) Measured $I_{\mathrm{s}}^{\mathrm{LNP}}/I_{\mathrm{s}}^{\mathrm{Si}}$ values plotted versus n_{m} for each size interval, as defined in (*A*) and (*B*). A negative trend implies that the LNPs have a refractive index below that of the silica reference particles ($n_{\mathrm{Si}} = 1.44$) and vice versa.

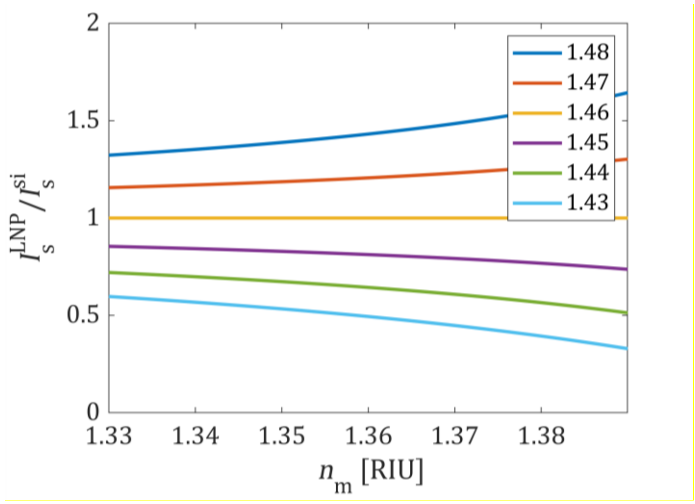

Fig. S8. The intensity ratio of LNPs and the reference silica particles as a function of the medium refractive index, $\boldsymbol{n}_{\mathbf{m}}$, where the value of $\boldsymbol{n}_{\mathbf{LNP}}$ is stepwise varied between 1.43 and 1.48.

10. Atto-488 labelled DMPE-PEG LNPs

In analogy to our theoretical treatment of scattering intensity in the main text and in Sect. 4 of the Supporting Information, the fluorescence emission from a spherical particle of radius r_{LNP} with fluorophores homogenously distributed within its volume can be expressed as

$$I_{\mathrm{f}} = I_{\mathrm{f0}}\eta_{\mathrm{ev,f}}\,, \tag{S11}$$

where $I_{\mathrm{f0}} \propto r_{\mathrm{LNP}}{}^{3}$ and

$$\eta_{\mathrm{ev,f}} = 3\left(\frac{\delta}{r_{\mathrm{LNP}}}\right)^{3} \exp\left(-\frac{r_{\mathrm{LNP}}}{\delta}\right)\left[\frac{r_{\mathrm{LNP}}}{\delta}\cosh\left(\frac{r_{\mathrm{LNP}}}{\delta}\right) - \sinh\left(\frac{r_{\mathrm{LNP}}}{\delta}\right)\right] \tag{S12}$$

If fluorophores are homogenously distributed on the outer surface of the spherical particle the expressions change to $I_{\mathrm{f0}} \propto r_{\mathrm{LNP}}{}^{2}$ and

$$\eta_{\mathrm{ev,f}} = \left[1 - \exp\left(-\frac{2r_{\mathrm{LNP}}}{\delta}\right)\right] {}^{\delta}\!/_{2r_{\mathrm{LNP}}}\,. \tag{S13}$$

If one instead assumes a particle with fluorophores homogenously distributed within its volume and a non-fluorescent outer surface, with a shell-thickness t the expression given in Eqs. S11-S12 change to $I_{\mathrm{f0}} \propto (r_{\mathrm{LNP}} - t)^{3}$ and

$$\eta_{\mathrm{ev,f}} = 3\left(\frac{\delta}{r_{\mathrm{LNP}}}\right)^{3} \exp\left(-\frac{r_{\mathrm{LNP}}}{\delta}\right)\left[\frac{r_{\mathrm{LNP}}}{\delta}\cosh\left(\frac{r_{\mathrm{LNP}}}{\delta}\right) - \sinh\left(\frac{r_{\mathrm{LNP}}}{\delta}\right)\right] +$$
$$3\frac{\delta^{2}t}{r_{\mathrm{LNP}}{}^{3}}\exp\left(-\frac{r_{\mathrm{LNP}}}{\delta}\right)\left[2\cosh\left(\frac{r_{\mathrm{LNP}}}{\delta}\right) - \frac{2r_{\mathrm{LNP}}}{\delta}\sinh\left(\frac{r_{\mathrm{LNP}}}{\delta}\right) - 2\right] + O(t)^{2} \tag{S14}$$

In Fig. S9*A* we have plotted $\eta_{\mathrm{ev,f}}$ as function of r_{LNP} for the specific wavelengths used in our measurements and for different non-fluorescent shell-thicknesses t according to Eq. S14. The correction factors differ substantially for different wavelengths but the variation between different shell-thicknesses for each specific wavelength is neglectable. One can thus safely use the simple expression given in Eq. S12 as a correction factor for LNPs irrespective of non-fluorescent shell-thicknesses below 15 nm, which we can assume is the case for the LNPs used in our experiments.

In Fig. S9*B* we present the measured fluorescence intensities ($I_{\mathrm{f}}/\eta_{\mathrm{ev,f}}$) for Cy5-mRNA and Atto-488-PEG versus their corresponding measured scattering intensities (I_{s}/η_{s}) with linear fits to the data after removing outliers (low Cy5-mRNA fluorescence LNPs). The fitted lines have slopes of 0.55 and 0.34 for Cy5-mRNA and Atto-488-PEG respectively, thus confirming that the Cy5-mRNA dye is distributed within the volume of the LNPs while the Atto-488 dye is confined to the surface of the LNPs.

It should be noted that the size distribution of the Atto-488 labelled DMPE-PEG LNPs was not evaluated using the methodology of changing the refractive index of the medium surrounding the LNPs as described in the main text and applied for the low- and high-DSPC LNPs. Instead, the sixth-root of the scattering intensity from each of the Atto-488 labelled DMPE-PEG LNPs was aligned to the size distribution of the LNPs obtained using NTA using the following relation:

$$r \approx \frac{\bar{r}_{\mathrm{NTA}}}{\overline{I_{\mathrm{s}}^{1/6}}} I_{\mathrm{s}}^{1/6}, \tag{S15}$$

where $\bar{r}_{\mathrm{NTA}}$ and $\overline{I_{\mathrm{s}}^{1/6}}$ are the median of the size distribution obtained with NTA and the median of the sixth root of the scattering intensity, obtained by the waveguide, respectively. For each scattering intensity, η_{s} was then calculated using Eqs. S4 and S5.

In Fig. S9*C* we have plotted the fluorescence intensities against each other and a fitted line through the data neglecting outliers. The resulting fitted line has a slope of 1.5, indicating the different spatial distribution of the two fluorescent dyes.

Figure S9*D* shows the anticipated fluorescence intensities of Cy5-mRNA and Atto-488-PEG obtained from Eqs. S11-S13 versus measured scattering intensity divided by the correction factor η_{s} from Eqs. S4-S5. The Cy5-mRNA fluorescence is calculated assuming homogenous distribution of the fluorophore within a volume, $\frac{4\pi}{3}(r_{\mathrm{LNP}} - t)^{3}$ and plotted for different values of the non-fluorescent shell-thickness, t (red datapoints) with fitted lines and their respective slopes indicated in the legend. The Atto-488-PEG fluorescence is calculated assuming homogenous distribution of the fluorophores on the surface area, $4\pi r_{\mathrm{LNP}}{}^{2}$, of the LNPs (blue datapoints) with a fitted line and its slope indicated in the legend.

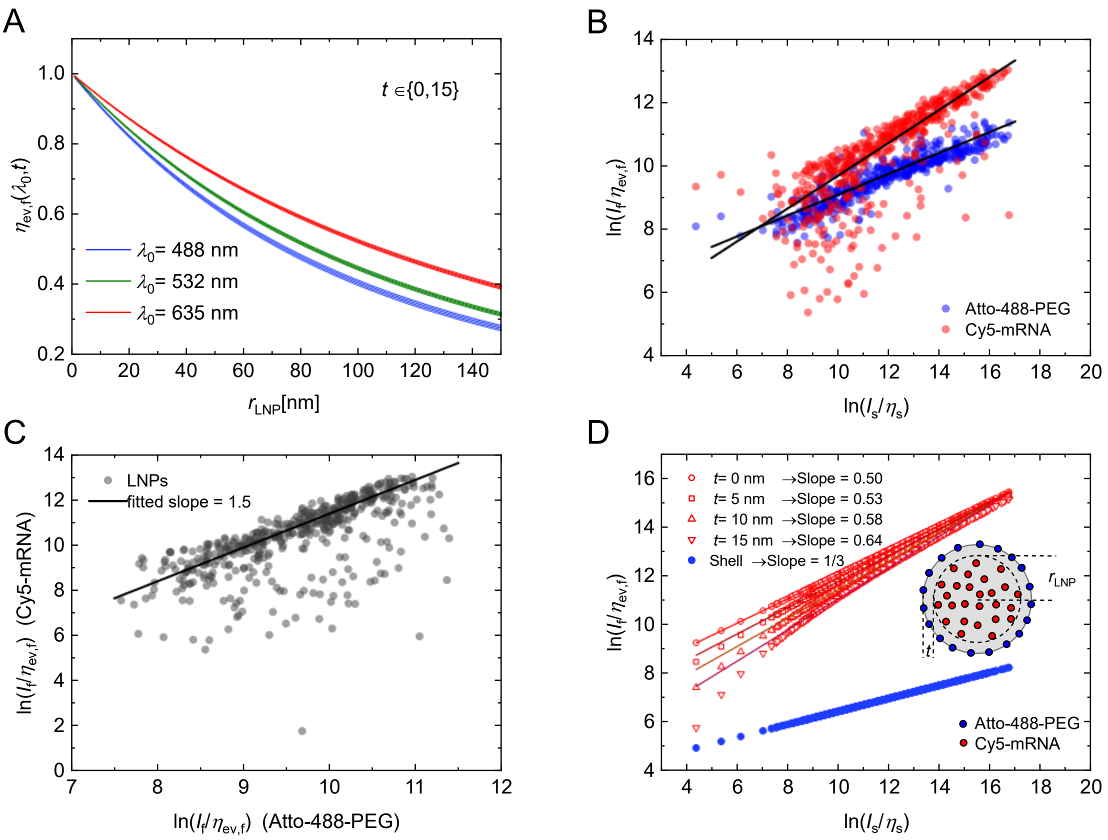

Fig. S9. (*A*) Dimensionless fluorescence correction-factors $\eta_{\text{ev,f}}(\lambda_0, t)$ according to Eq. S12 for the three wavelengths used in our study and for various non-fluorescent shell thicknesses t. (*B*) Logarithmic plots of fluorescence $\left(I_\text{f}/\eta_{\text{ev,f}}\right)$ and scattering $(I_\text{s}/\eta_\text{s})$ intensities from LNPs containing 20% labelled Cy5-mRNA (semi-transparent spheres of color red) and 50% Atto-488 labelled DMPE-PEG lipids (semi-transparent spheres of color blue) with lines fitted through the data, neglecting outliers from LNPs with low Cy5-mRNA intensities. The lines have a slope of 0.55 and 0.34 for Cy5-mRNA and Atto-488-PEG, respectively, indicating that the Atto-488-PEG-lipid intensities scale with the area of the LNPs ($I_f \propto {r_{\text{LNP}}}^2 \propto {I_\text{s}}^{1/3}$), while the Cy5-mRNA intensities scale with the volume ($I_\text{f} \propto {r_{\text{LNP}}}^3 \propto {I_\text{s}}^{1/2}$). (*C*) Measured Cy5-mRNA fluorescence intensities versus measured Atto-488-PEG fluorescence intensities with a line fitted through the data, neglecting outliers from LNPs with low Cy5-mRNA intensities. The line has a slope of 1.5 confirming the different spatial distribution of the two labels in/on the LNPs. (*D*) Anticipated fluorescence intensity curves versus measured scattering intensities assuming Atto-488 labelled PEG is distributed in a shell-like configuration in accordance with Eqs. S11 and S13 (blue spheres in schematic) and the Cy5-labelled mRNA is evenly distributed in a core of radius $r_{\text{LNP}} - t$ for $t = 0, 5,$ 20 and 15 nm (red spheres in schematic) in accordance with Eqs. S11 and S12.

11. Cy5-mRNA and Rhod-DOPE labelled LNPs

Figures S10*A* and S10*C* show the anticipated fluorescence intensities as a function of measured radii, r_{LNP}, for low-DSPC and high-DSPC LNPs, respectively. The fluorescence intensity values are calculated using Eqs. S11-S12 for different values of t and a line fitted through the data with respective slopes indicated in the legend of the figures. The figures show that the anticipated slope is expected to increase with increasing thickness of nonfluorescent shell thickness t, from 3, for when the fluorescent material occupies the entire volume of the particles ($t = 0$), to around 3.8 when the fluorescent material is confined to an inner core of radius $r_{\text{LNP}} - 15$.

Figures S10*B* and S10*D* show the anticipated Cy5-mRNA versus Rhod-DOPE fluorescence intensities for low- and high-DSPC LNPs, respectively, when assuming that the Rhod-DOPE signal originates from the entire volume of the particle (r_{LNP}) while the Cy5-mRNA signal originates from an inner core of radius $r_{\text{LNP}} - t$, with a line fitted through the data with respective slopes indicated in the legends. The slopes decrease with increasing shell thickness, from 1 (for t =0) to 0.8 (for $t = 15$ nm).

The anticipated slopes in Fig. S10 should be compared to the measured slopes presented in Fig. 3 of the main text.

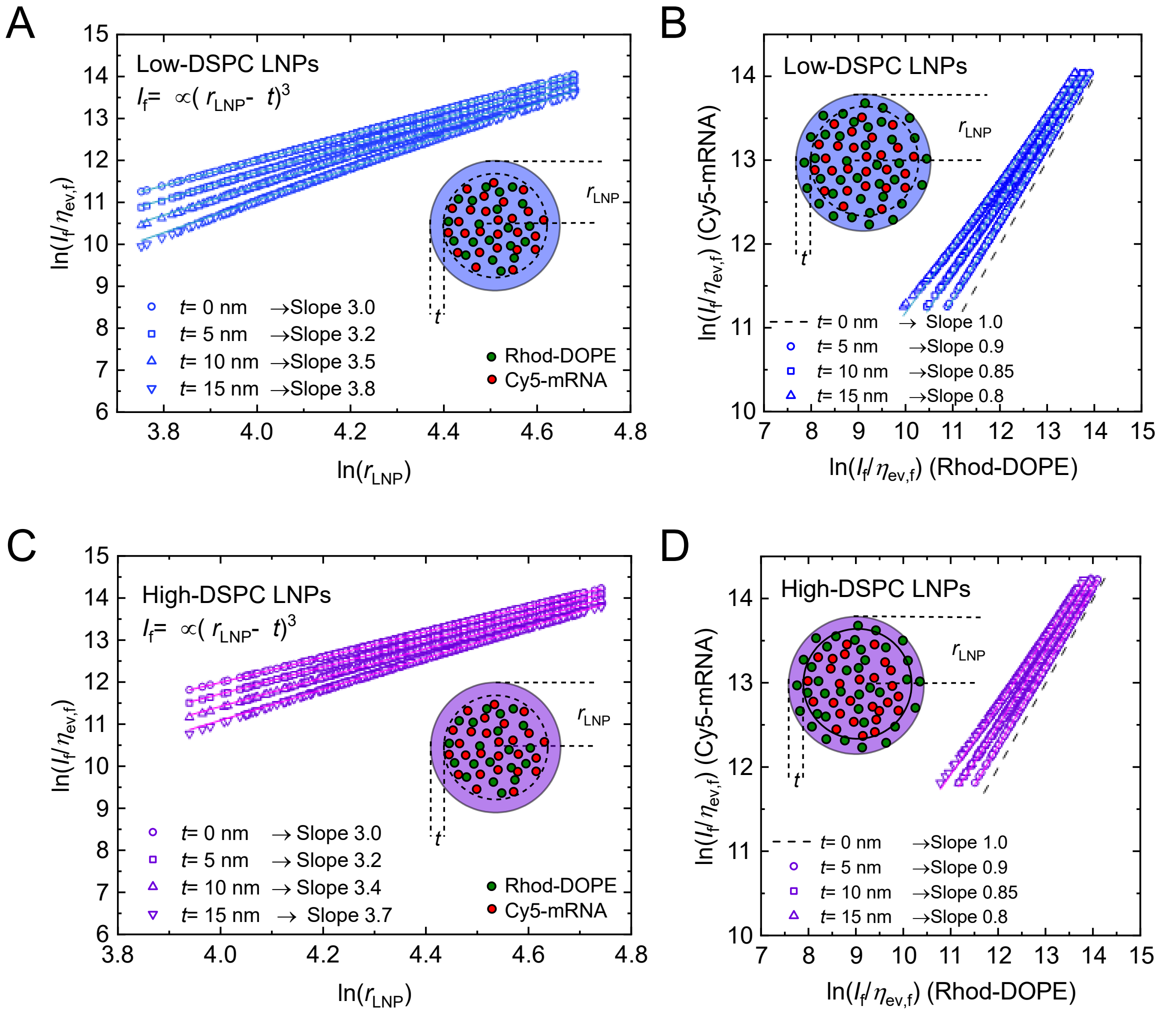

Fig. S10. Estimated fluorescence intensities for (*A*) low- and (*C*) high-DSPC LNPs for different values of nonfluorescent shell-thicknesses t, with lines fitted through the data and their respective slopes displayed in the legend. Cy5-mRNA (indicated by red spheres in the inset-schematic) and Rhod-DOPE (indicated by green spheres in the inset-schematic) are assumed to occupy the same spherical volume of radius $r_{\mathrm{LNP}} - t$. (*B*) and (*D*) display the Cy5-mRNA fluorescence intensity versus Rhod-DOPC fluorescence intensity for (*B*) low-DSPC LNPs and (*D*) high-DSPC LNPs for various shell-thicknesses t. Rhodamine is assumed to scale with a spherical volume of radius r_{LNP} while the Cy5 scales with the spherical volume of radius $r_{\mathrm{LNP}} - t$. Lines are fitted through the data and their respective slopes indicated in the legend.

12. Cy5-mRNA, Rhod-DOPE, refractive index and fusogenicity of LNPs

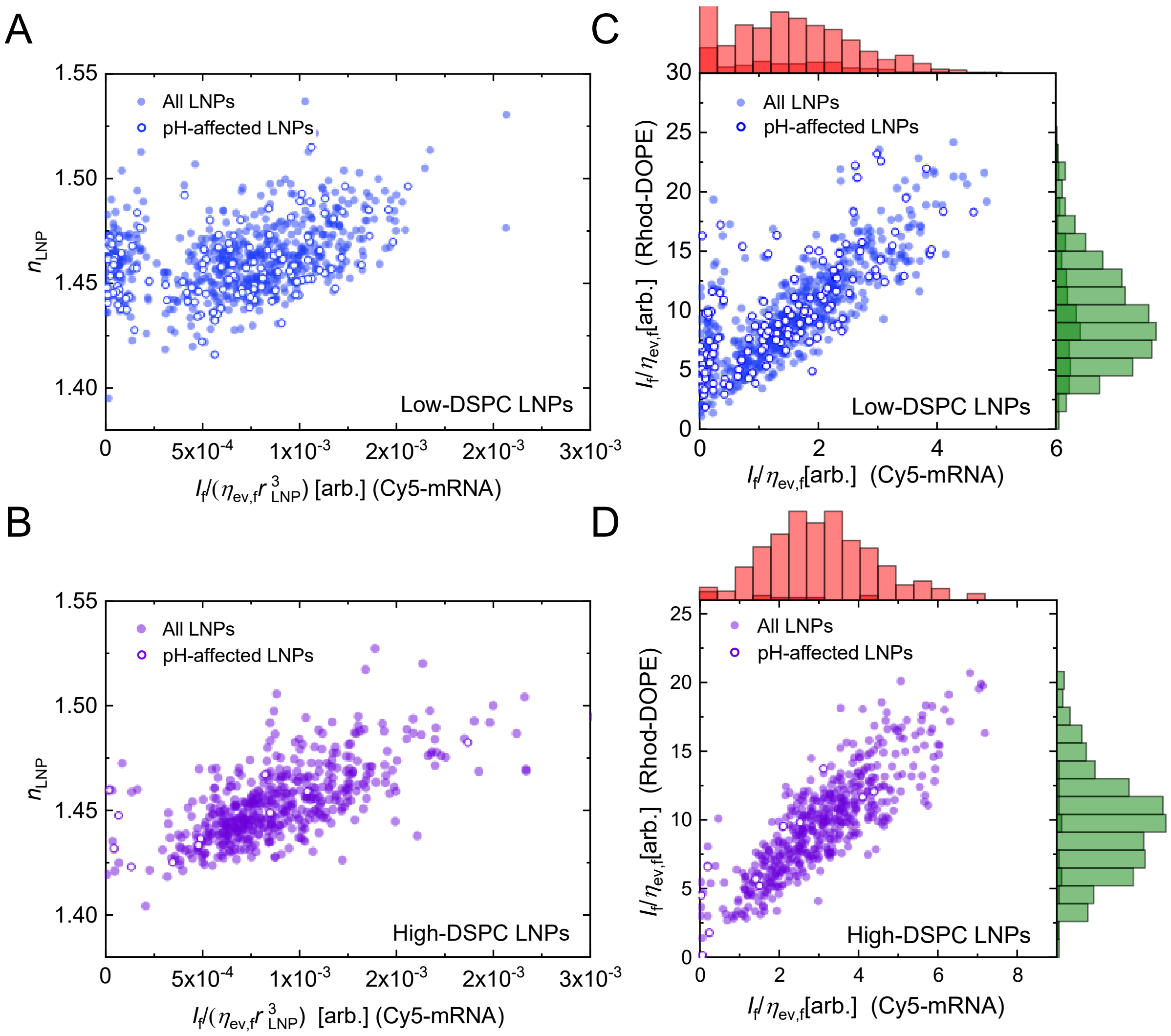

Fig. S11. Graphs highlighting two main observations: i) the correlation between measured quantities within the subpopulation of LNPs displaying diminishing Cy5-mRNA fluorescence intensities and ii) the correlation between measured quantities of LNPs that undergo fusion (hollow circles) for low-DSPC LNPs [(*A*) and (*C*)] and high-DSPC LNPs[(*B*) and (*D*)]. (*A*) and (*B*) show a general positive correlation between refractive index of the LNPs and their Cy5-mRNA content per volume, but no correlation between LNPs displaying diminishing Cy5-mRNA intensities and refractive index. Note how the LNPs with diminishing Cy5 intensities display a broad distribution in refractive indices, spanning the same refractive index values as the brighter LNPs. (*C*) and (*D*) indicate a general positive correlation between Rhodamine and Cy5 fluorescence but no correlation between Rhodamine intensities and Cy5 fluorescence intensities for LNPs exhibiting diminishing Cy5 intensities. Open circles represent LNPs that undergo fusion with the supported lipid bilayer to which they are tethered upon pH rapid reduction in pH from 7.4 to 6.0. No correlation is observed between LNP tendency to fuse and its corresponding fluorescence intensity (Cy5-mRNA or Rhod-DOPE) or refractive index.

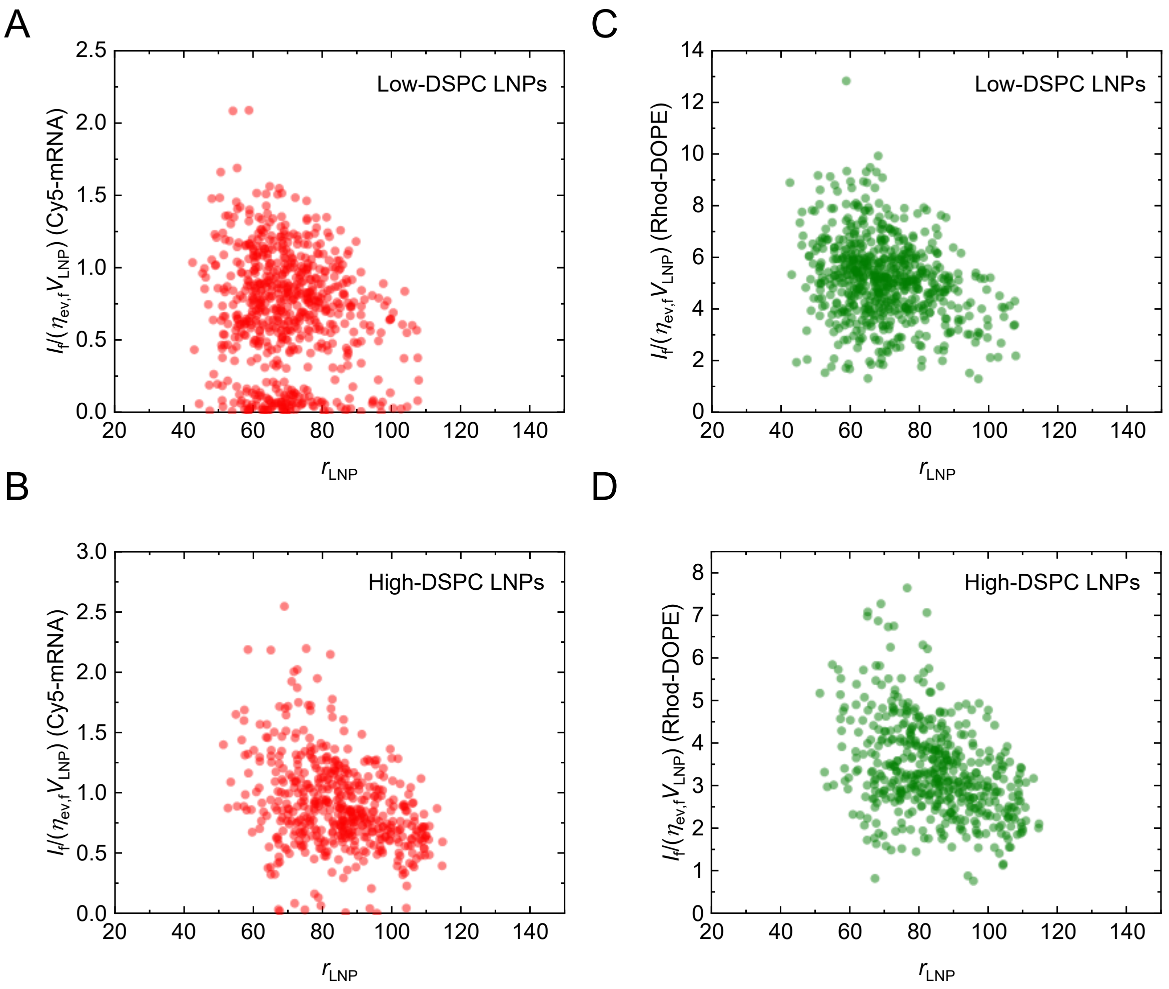

Fig. S12 Measured fluorescence intensity per LNP volume (Cy5-mRNA in red and Rhod-DOPE in green) for low-DSPC [(*A*) and (*C*)] and high-DSPC [(*B*) and (*D*)]. Data in (*A*) and (*B*) are the same as presented in Fig. 4 in the main text. The data seems to indicate negative correlation between fluorescence per volume and LNP size but this trend is more likely an artifact due to uncertainties in LNP size (r_{LNP}) estimation (see corresponding text in the current section and Fig. S14).

The effective refractive index (n_{eff}) of an LNP (or a solute mixture) can be evaluated from the Lorentz-Lorenz equation and the Bruggeman effective refractive index evaluation formula:

$$\sum_i f_i \frac{n_{\mathrm{eff}} - n_i}{n_{\mathrm{eff}} + 2n_i} = 0\,, \qquad (S16)$$

where n_i and f_i represents the refractive index and volume fraction of constituent i, respectively.

Assuming a typical LNP is made from three main components; water ($n = 1.334$), lipids ($n = 1.48$) and mRNA ($n = 1.6$), the effective refractive index can be evaluated for different volume ratios. In Fig. S13 we plot n_{eff} as a function of mRNA content for an LNP with varying water content. The span of the n_{eff} for a fixed water content but mRNA content varying between 5-20% is approximately 0.015 refractive-index units. Assuming that Cy5-mRNA content scales with mRNA content (which is rational) a 5-20% variation (four-fold variation) in mRNA content should result in a 4-fold variation in Cy5-mRNA fluorescence intensity, which is close to what is observed in our measurements (see Fig. S11). The corresponding effect on n_{eff} for a fixed mRNA content but varying water content between 20-30% is also approximately 0.015 refractive index units, with LNPs consisting of only lipids, and no water or mRNA, having a refractive index of $n_{\mathrm{eff}} = 1.48$.

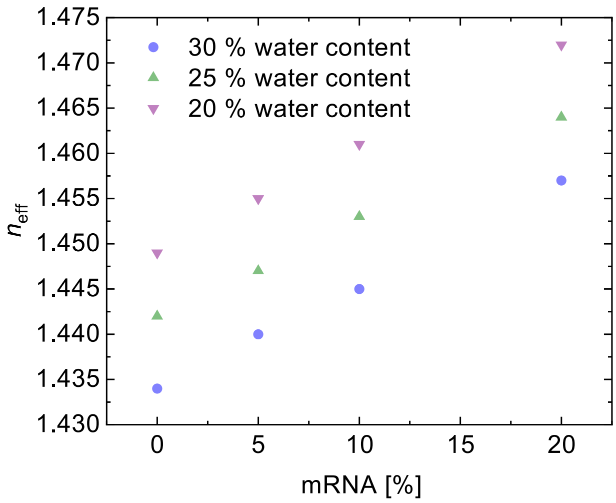

Fig. S13 LNP effective refractive index, calculated using Eq. S16, versus mRNA content for different water contents.

The plots for measured Cy5-mRNA and Rhod-DOPE fluorescence intensities per volume versus LNP radius for low- (Figs. S9*A* and S9*C*) and high-DSPC (Figs. S9*B* and S9*D*) LNPs seem to indicate a general tendency for the smaller LNPs to display a higher fluorescence intensity per volume than the larger LNPs. However, we believe this to be an artifact related to uncertainties in size determination as elaborated on below.

To determine the radius of an LNP (r_{LNP}) we adopt an analysis strategy that is entirely independent of the LNP's fluorescence intensity (I_{f}) (see main text). This means that errors or uncertainties in fluorescence intensity determination (ΔI_{f}) are uncorrelated to errors or uncertainties in size determination (Δr_{LNP}).

This scenario can be simulated (here done in Matlab) in the following manner: First, we note that our LNPs have a log-normal size distribution. Such a distribution can be characterized by a log-normal mean (μ_{Ln}) and log-normal standard deviation (σ_{Ln}) which are related to the real mean (μ) and real standard deviation (μ) according to:

$$\mu_{\mathrm{Ln}} = \mathrm{Ln}\left[\frac{\mu^2}{\sqrt{\sigma^2 + \mu^2}}\right] \quad (S17)$$

$$\sigma_{\mathrm{Ln}} = \sqrt{\mathrm{Ln}\left[\frac{\sigma^2}{\mu^2} + 1\right]} \quad (S18)$$

Then we identified the real mean (`r_0`), and standard deviation (`sigma_r_0`) of our measured LNPs and simulated set of LNPs (`r`) with same size distribution and same number of LNPs (`N`) using Eqs. S17 and S18 and the built-in log-normal random number generator function in Matlab (`lognrnd`).

```
mu_r = log(r_0^2./sqrt(sigma_r_0.^2+r_0.^2));
sigma_r= sqrt(log( sigma_r_0.^2./r_0.^2 + 1));
r = lognrnd(mu_r,sigma_r,[1,N]);
```

To calculate the fluorescence intensity per LNPs (`If`) we first calculated the expected fluorescence value (`I_f_0`) according to $I_{\mathrm{f0}} \propto (r_{\mathrm{LNP}} - t)^3$ for non-fluorescent shell thicknesses $t = 0, 5, 10$ and 15 nm. We furthermore defined an uncertainty (`Delta_I_f`) of the expected value of 10%.

```
I_f_0 = (r-t).^3;
Delta_I_f = 0.1.*I_f_0;
```

For each LNP we then assigned the fluorescence intensity (`If`) by randomly picking a value from a log-normal distribution with lognormal mean (`mu_I_f`) and lognormal standard deviation (`sigma_I_f`) according to Eqs. S17 and S18.

```
mu_I_f = log(I_f_0.^2./sqrt(std_I_f.^2+I_f_0.^2));
```

```
sigma_I_f = sqrt( log( std_I_f.^2./I_f_0.^2 +1));
If = lognrnd(mu_I_f,sigma_I_f);
```

We then added an 0-10% uncertainty (`Delta_percentage`) to the size of our LNPs (`r_2`). This uncertainty is totally uncorrelated to the uncertainty in fluorescence (`sigma_I_f`) and was obtained by randomly picking a value from a log-normal distribution with lognormal mean (`mu_r_2`) and lognormal standard deviation (`sigma_I_f`) according to Eqs. S17 and S18.

```
Delta_percentage = 0.15;
Delta_r_2 = Delta_percentage.*r;
mu_r_2 = log(r.^2./sqrt(Delta_r_2.^2+r.^2));
sigma_r_2= sqrt(log(Delta_r_2.^2./r.^2 + 1));
r_2 = lognrnd(mu_r_2,sigma_r_2);
```

Finally a vector with our simulated data (`r_2,If./(r_2.^3)`) was generated and plotted as shown in Fig. S14**.**

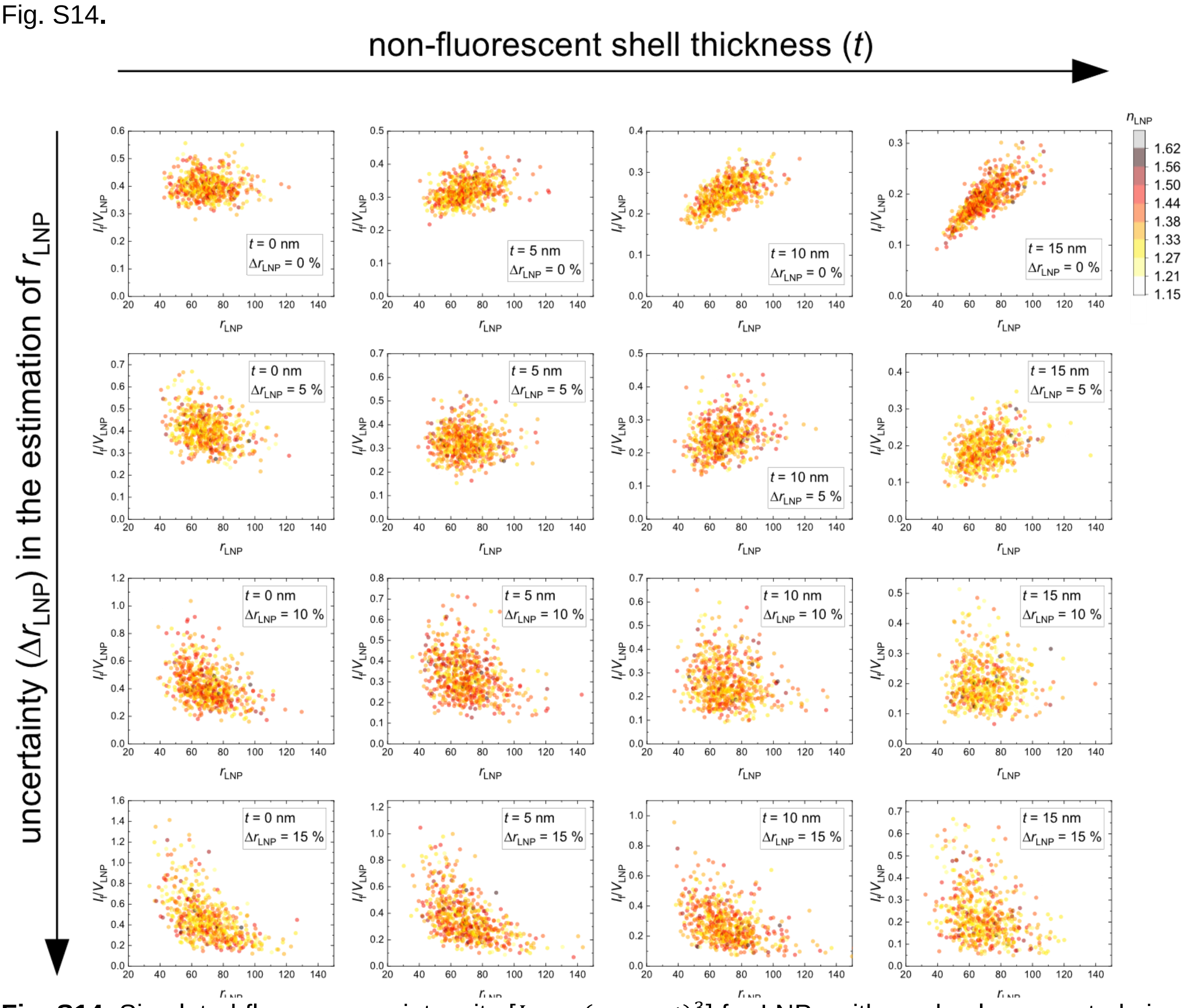

Fig. S14. Simulated fluorescence intensity [$I_{f0} \propto (r_{LNP} - t)^3$] for LNPs with randomly generated sizes with similar size-distribution as obtained for the measured low-DSPC LNPs. The plots are arranged on x- and y-axes corresponding to increased non-fluorescent shell thickness (t) and increased uncertainty (Δr_{LNP}) in estimation of r_{LNP}, respectively. Errors in size estimation (Δr) and fluorescence intensities (ΔI_f) are uncorrelated and expressed as percentage of r_{LNP} and I_{f0}, respectively. Uncertainty in fluorescence intensities is kept constant at $\Delta I_f = 10\%$.

13. LNP refractive index distributions

Even though it was concluded in the main text that some of the refractive index variation across LNP size shown in Fig. 2 (main text) is due to measurement uncertainty, it is worthwhile to note that the data in Fig. 4 (main text) suggest a decrease in refractive index with Cy5-mRNA fluorescence intensity per unit volume for both types of LNPs. This could potentially be due to the lower polarizability (refractive index) of lipids than mRNA[9]. To estimate the magnitude of such a variation, it is instructive to recall that the average LNP contains 65% lipid, 10% mRNA and 25% water[10], with mRNA being expected to reside in water-compartments within the LNPs [9], [10], [11], [12]. Assuming that for the low-Cy5-mRNA-fluoresent subpopulation, the mRNA is replaced by lipid material, with no change in water content, an LNP consisting of 75% lipid material and 25% water, will exhibit a difference in refractive index of ~0.02 (see Supporting Information, Sect. 11, Fig. S13), which is similar to the observed spread in refractive index (Supporting information Sect. 8). However, considering that the subpopulation of LNPs with low or negligible amounts of mRNA displays a similar size and refractive index distribution as LNPs containing Cy5-mRNA (Fig. 4 and Figs. S11 and S13 in Supporting Information), and that also Rhod-DOPE display a very similar trend (see Supporting Information, Sect. 11, Fig. S12), we conclude that the observed refractive index variation is most likely attributed to a variation in water content, rather than mRNA content, across LNP size.

14. LNP uptake and hEPO expression in primary human adipocytes and iPSC-derived human hepatocytes

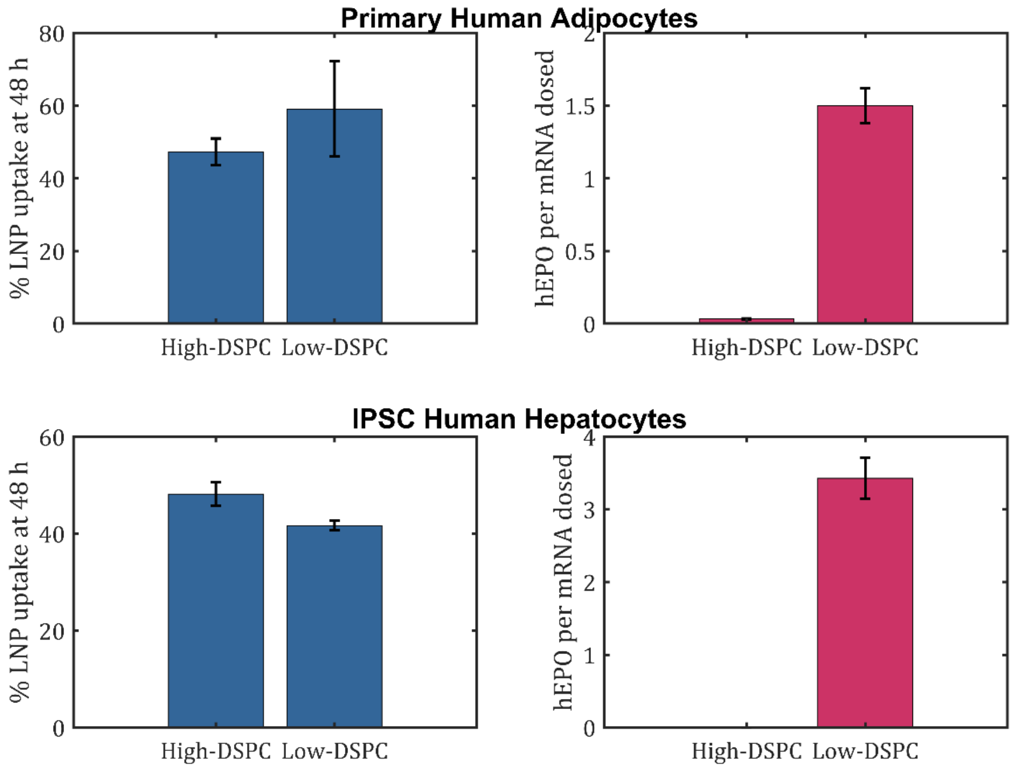

Fig. S15. LNP uptake and human erythropoietin (hEPO) expression in primary human adipocytes and iPSC-derived human hepatocytes. (A, C) Percentage of LNP uptake at 48 h and (B, D) number of hEPO proteins expressed per mRNA dosed at 48 h for high-DSPC (blue) and low-DSPC (red) LNPs. (A, B) Data for primary human adipocytes. (C, D) Data for iPSC-derived human hepatocytes. Values represent means ± SEM (n = 3). The experiments were conducted in the presence of 1% human serum. Parts of this data has been published previously[13]

15. Comparing fusogenic and non-fusogenic LNPs

In order to elucidate potential correlations between the measured physicochemical LNP characteristics and fusogenicity, the histograms in Fig S16 display LNP refractive index (A) and radius (B) for either the low-DSPC LNPs which, upon a medium pH decrease from 7.4 to 6, underwent fusion with the underlying SLB or those that did not. For the fused and non-fused populations, the median radii are 71.10 ± 13.43 nm and 68.38 ± 12.46 nm, respectively, while the corresponding refractive indices are 1.4559 ± 0.0175 and 1.4571 ± 0.0182. The close similarity in these values suggests that neither size nor refractive index is the primary determinant of fusogenicity.

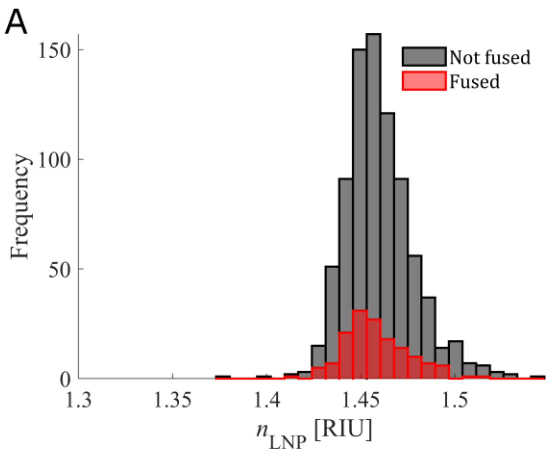

Fig. S16. Histograms comparing the refractive index (A) and radius (B) distributions of low-DSPC LNPs which either do (red) or do not (black) undergo fusion with the underlying supported lipid bilayer upon a decrease in pH from 7.4 to 6 for the surrounding medium.

16. Reversibility of refractive index buffer change

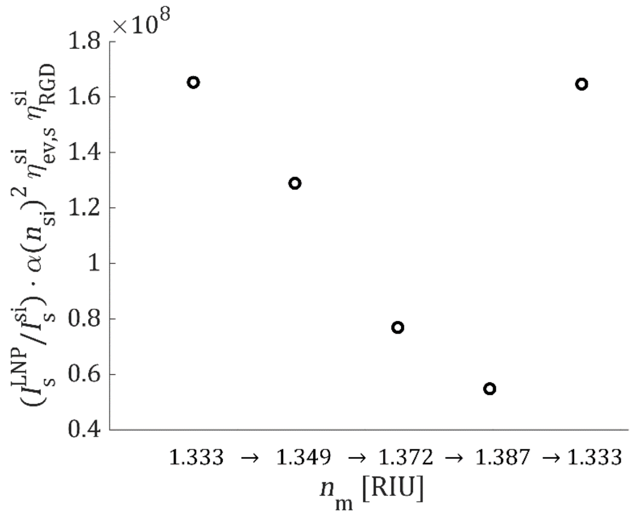

Fig. S17. The measured mean scattering intensity ratios for LNPs in a representative measurement where the medium refractive index n_m is sequentially increased from 1.333 to 1.387 and back to 1.333. As the medium is changed back to the initial PBS solution, the intensity returns to the initial value, implying that the LNPs are not irreversibly influenced when exposed to the iodixanol solutions.

17. Supporting movies

M1: Injection LNPs to streptavidin modified supporting lipid bilayer monitored using 488 nm light in scattering. The data was captured at a frame rate of 0.5 f/s (but the uploaded movie is sped up 50x), and field of view is approximately 100 µm.

M2: Low-DSPC LNPs, bound to a streptavidin modified supported lipid bilayer, as the surrounding solution is exchanged from PBS at pH 7.4 to PBS at pH 6. The data was captured at a frame rate of 0.5 f/s (but the uploaded movie is sped up 50x), and field of view is approximately 100 µm.

M3: High-DSPC LNPs, bound to a streptavidin modified supported lipid bilayer, as the surrounding solution is exchanged from PBS at pH 7.4 to PBS at pH 6. The data was captured at a frame rate of 0.5 f/s (but the uploaded movie is sped up 50x), and field of view is approximately 100 µm.

References

[1] C. M. Waterman *et al.*, "A tunable refractive index matching medium for live imaging cells, tissues and model organisms," 2017, doi: 10.7554/eLife.27240.001.

[2] D. Lee *et al.*, "High-fidelity optical diffraction tomography of live organisms using iodixanol refractive index matching," *Biomed Opt Express*, vol. 13, no. 12, p. 6404, Dec. 2022, doi: 10.1364/boe.465066.

[3] M. Mapar, M. Sjöberg, V. P. Zhdanov, B. Agnarsson, and F. Höök, "Label-free quantification of protein binding to lipid vesicles using transparent waveguide evanescent-field scattering microscopy with liquid control," *Biomed Opt Express*, vol. 14, no. 8, p. 4003, 2023, doi: 10.1364/boe.490051.

[4] C. F. Bohren and D. R. Huffman, *Absorption and scattering of light by small particles*. John Wiley & Sons, 1983.

[5] M. Hammer, "2-D mode solver for dielectric multilayer slab waveguides." [Online]. Available: http://www.computational-photonics.eu/oms.html

[6] C. Wang *et al.*, "Holographic Characterization of Protein Aggregates," *J Pharm Sci*, vol. 105, no. 3, 2016, doi: 10.1016/j.xphs.2015.12.018.

[7] A. D. Kashkanova, D. Albrecht, M. Küppers, M. Blessing, and V. Sandoghdar, "Measuring Concentration of Nanoparticles in Polydisperse Mixtures Using Interferometric Nanoparticle Tracking Analysis," *ACS Nano*, Jul. 2024, doi: 10.1021/acsnano.4c04396.

[8] B. Midtvedt *et al.*, "Fast and Accurate Nanoparticle Characterization Using Deep-Learning-Enhanced Off-Axis Holography," *ACS Nano*, vol. 15, no. 2, 2021, doi: 10.1021/acsnano.0c06902.

[9] M. J. Carrasco *et al.*, "Ionization and structural properties of mRNA lipid nanoparticles influence expression in intramuscular and intravascular administration," *Commun Biol*, vol. 4, no. 1, Dec. 2021, doi: 10.1038/s42003-021-02441-2.

[10] M. Y. Arteta *et al.*, "Successful reprogramming of cellular protein production through mRNA delivered by functionalized lipid nanoparticles," *Proc Natl Acad Sci U S A*, vol. 115, no. 15, pp. E3351–E3360, 2018, doi: 10.1073/pnas.1720542115.

[11] J. Viger-Gravel, A. Schantz, A. C. Pinon, A. J. Rossini, S. Schantz, and L. Emsley, "Structure of Lipid Nanoparticles Containing siRNA or mRNA by Dynamic Nuclear Polarization-Enhanced NMR Spectroscopy," *Journal of Physical Chemistry B*, vol. 122, no. 7, pp. 2073–2081, Feb. 2018, doi: 10.1021/acs.jpcb.7b10795.

[12] F. Sebastiani *et al.*, "Apolipoprotein E Binding Drives Structural and Compositional Rearrangement of mRNA-Containing Lipid Nanoparticles," *ACS Nano*, vol. 15, no. 4, pp. 6709–6722, Apr. 2021, doi: 10.1021/acsnano.0c10064.

[13] M. Y. Arteta *et al.*, "Successful reprogramming of cellular protein production through mRNA delivered by functionalized lipid nanoparticles," *Proc Natl Acad Sci U S A*, vol. 115, no. 15, pp. E3351–E3360, 2018, doi: 10.1073/pnas.1720542115.
